# Supplementary figures and images for: Connectome-based prediction of functional impairment in experimental stroke models
Source: PLoS One. 2024 Dec 19;19(12):e0310743. doi: 10.1371/journal.pone.0310743 (PMC11658581; doi:10.1371/journal.pone.0310743)

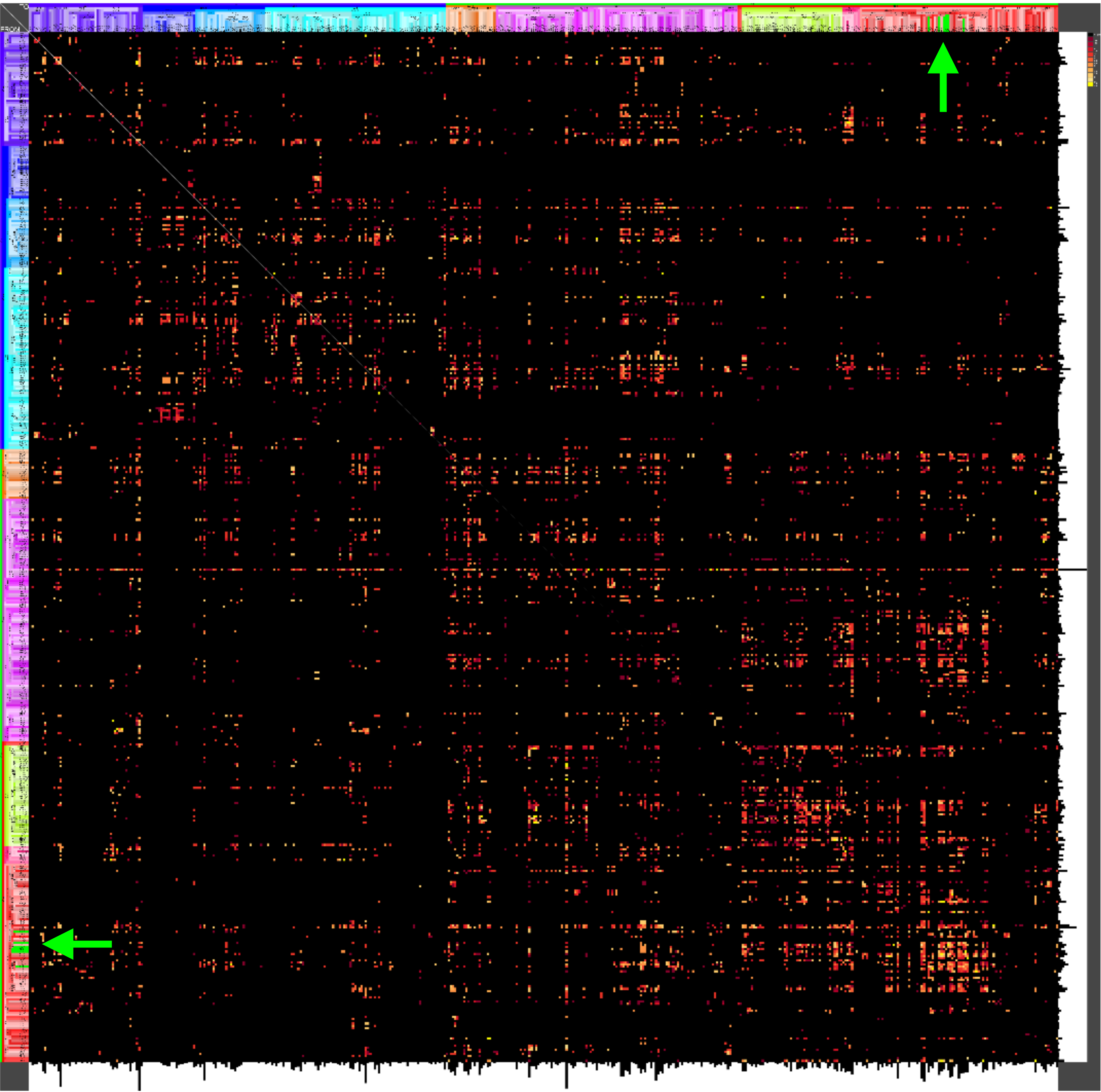

Supplement: S1 Fig — Cortical non-lesioned (red half-tones) and lesioned regions (arrow, green) are located in the upper right, resp., lower left part of the matrix. (PNG) [file pone.0310743.s001.png]

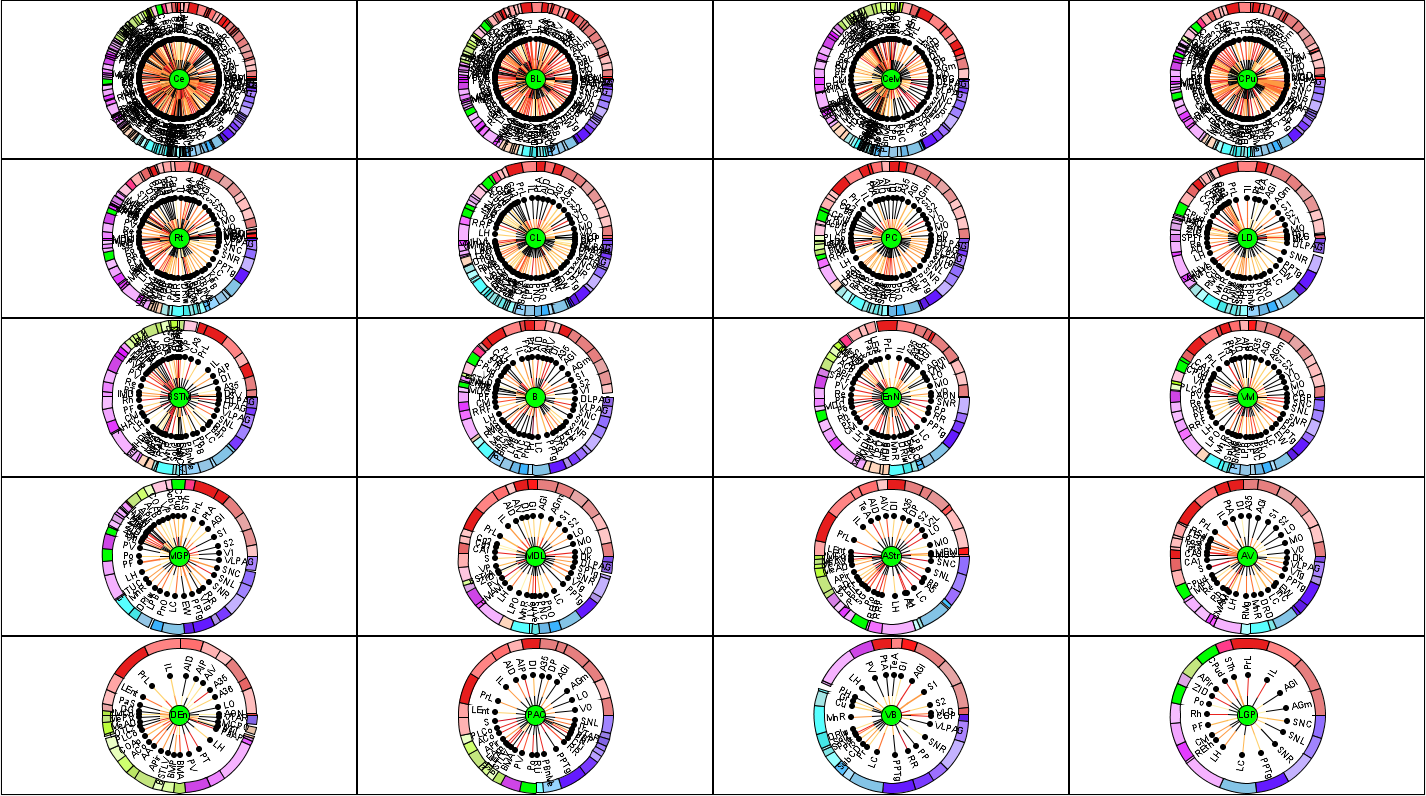

Supplement: S2 Fig — Distance from center region (lesioned region) is the average rank of local network parameters filtered for the 25% of lowest ranks (largest importance for the network). The lesioned regions were sorted with regard to the number of filtered and connected regions. All other lesioned regions have to large average ranks or too few connections and they are not displayed. (PNG) [file pone.0310743.s002.png]

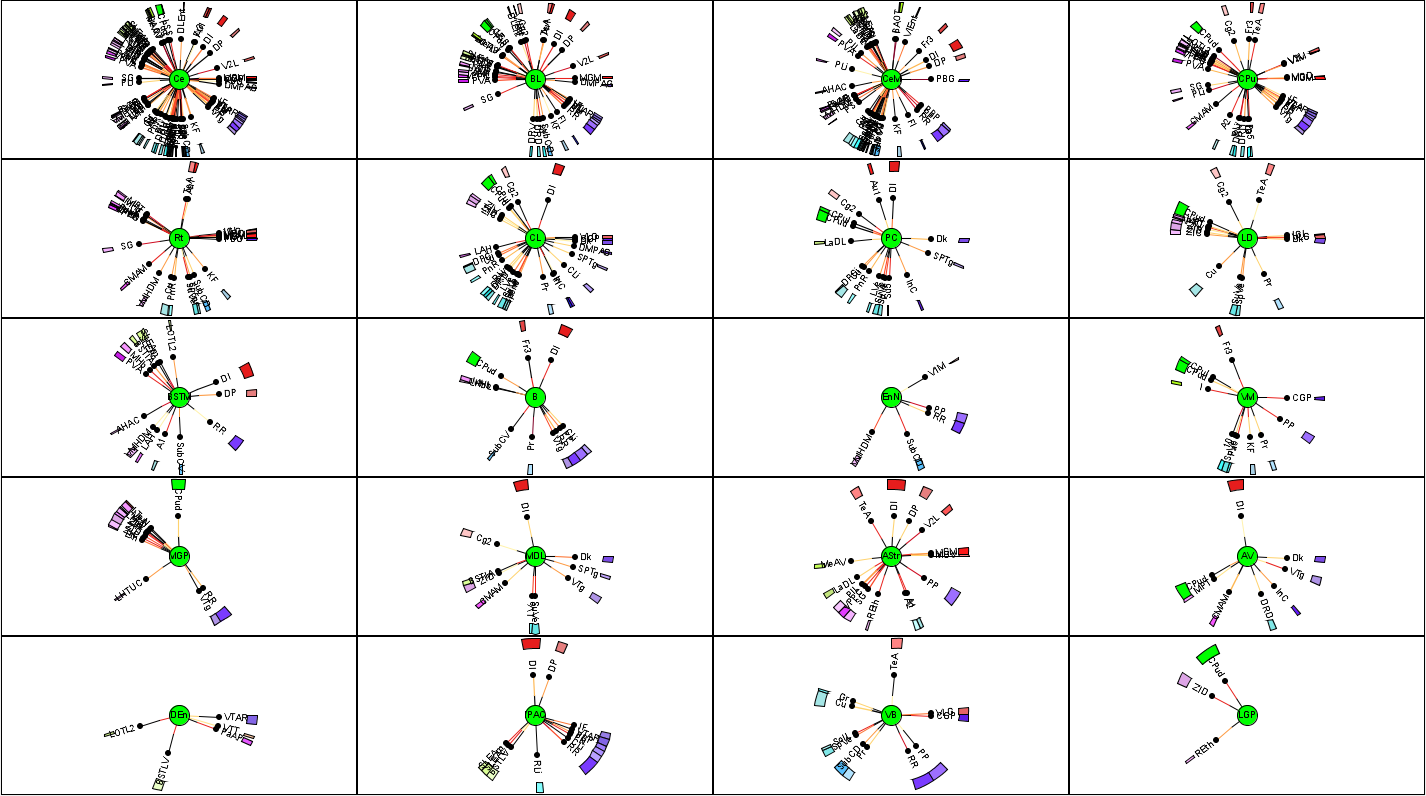

Supplement: S3 Fig — Distance from center region (lesioned region) is the average rank of local network parameters filtered for the 80% of lowest ranks (largest importance for the network). The lesioned regions were sorted with regard to the number of filtered and connected regions. (PNG) [file pone.0310743.s003.png]

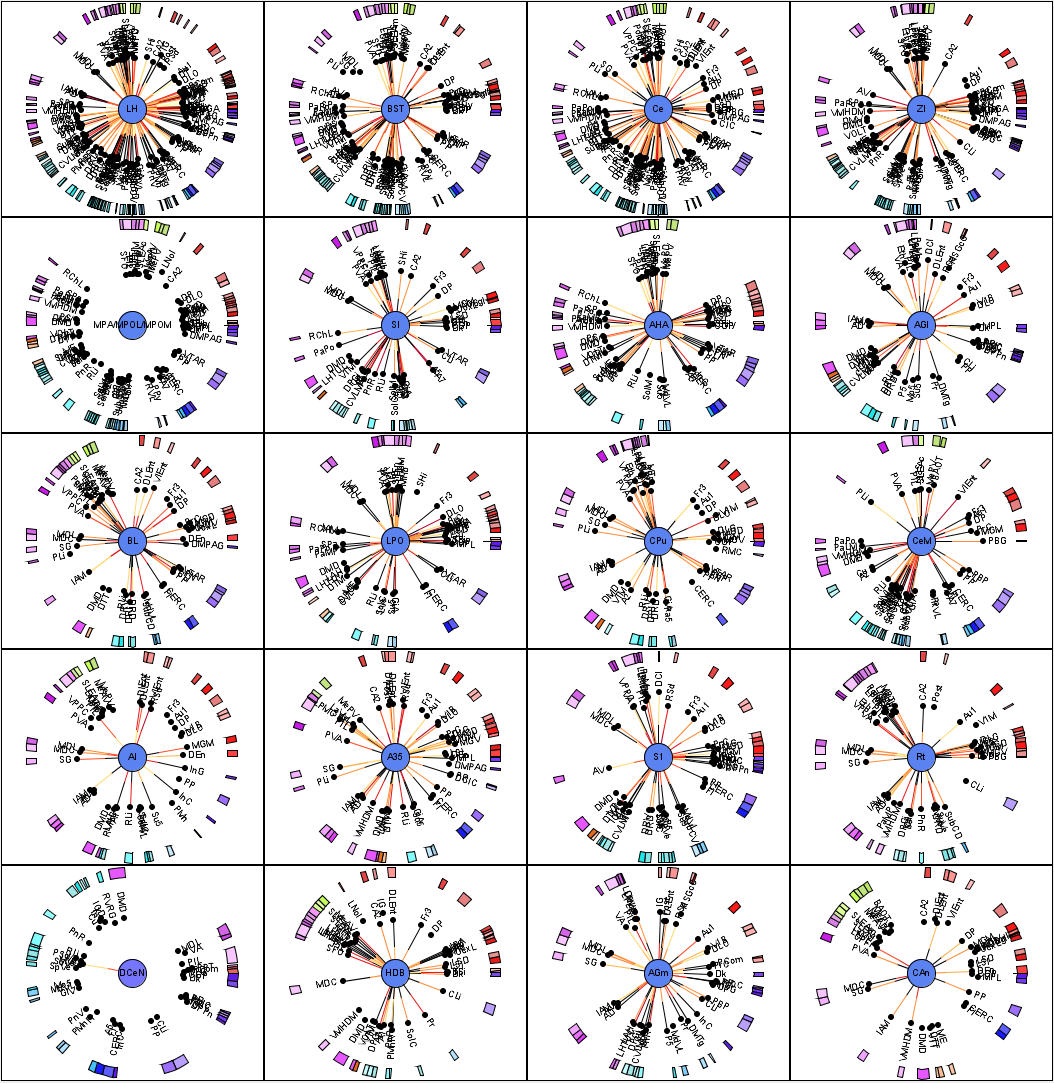

Supplement: S4 Fig — Filtering conditions are the same as described in Fig 11. (PNG) [file pone.0310743.s004.png]

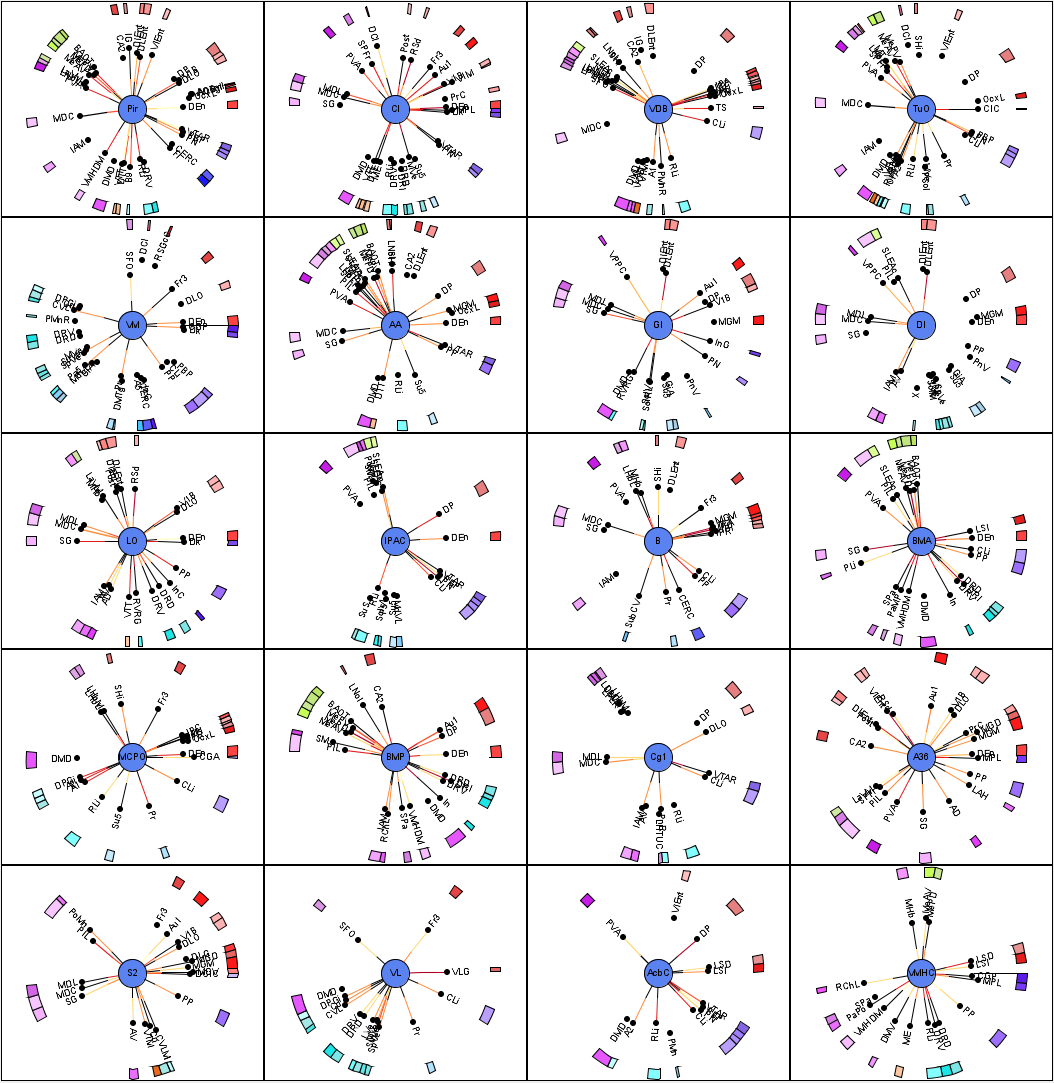

Supplement: S5 Fig — Filtering conditions are the same as described in Fig 11. (PNG) [file pone.0310743.s005.png]

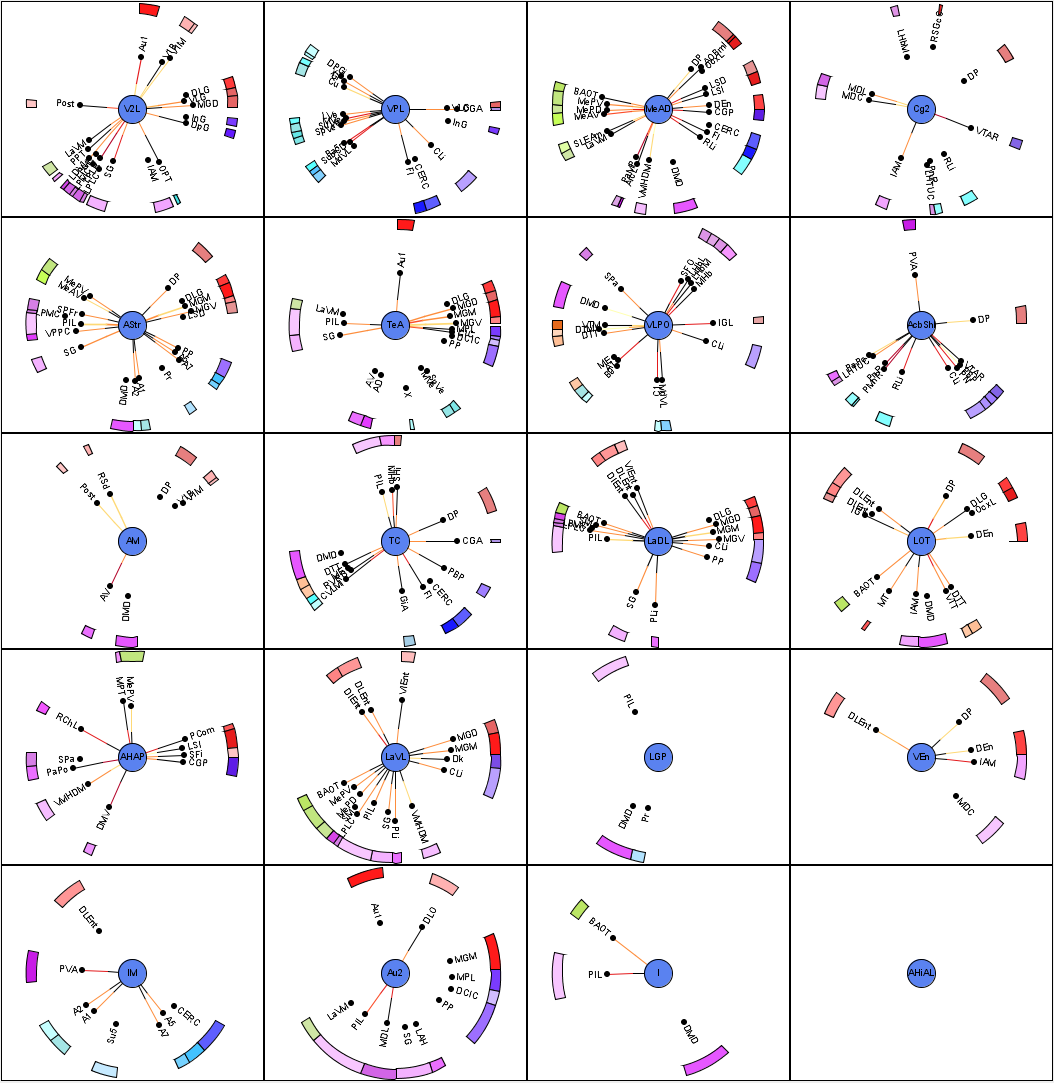

Supplement: S6 Fig — Filtering conditions are the same as described in Fig 11. (PNG) [file pone.0310743.s006.png]
