# Supplementary material for: Connectome-based prediction of functional impairment in experimental stroke models
Source: PLoS One. 2024 Dec 19;19(12):e0310743. doi: 10.1371/journal.pone.0310743 (PMC11658581; doi:10.1371/journal.pone.0310743)
Supplement: S1 Table — (PDF) [file pone.0310743.s007.pdf]

S1 Appendix

S0 Table: List of abbreviations.

**4**	Trochlear nucleus
**6**	Abducens nucleus
**10**	Dorsal motor nucleus of vagus
**12Sprin**	Principal hypoglossal nucleus
**3PC**	Oculomotor nucleus parvicellular part
**5Sol**	Trigeminal solitary transition zone
**7DI**	Facial nucleus dorsal intermediate subnucleus
**7DL**	Facial nucleus dorsolateral subnucleus
**7DM**	Facial nucleus dorsomedial subnucleus
**7L**	Facial nucleus lateral subnucleus
**7VI**	Facial nucleus ventral intermediate subnucleus
**7VM**	Facial nucleus ventromedial subnucleus
**A1**	A1 noradrenergic cells
**A11**	A11 dopamine cells
**A13**	A13 dopamine cells
**A2**	A2 noradrenergic cells
**A35**	Perirhinal cortex
**A36**	Ectorhinal cortex
**A5**	A5 noradrenaline cells
**A7**	A7 noradrenaline cells
**AA**	Anterior amygdaloid area
**AcbC**	Accumbens nucleus core
**AcbShl**	Lateral accumbens shell
**ACo**	Anterior cortical amygdaloid nucleus
**AD**	Anterodorsal thalamic nucleus
**AGl**	Lateral agranular prefrontal cortex
**AGm**	Medial agranular prefrontal cortex
**AHAA**	Anterior hypothalamic area anterior part
**AHAC**	Anterior hypothalamic area central part
**AHAP**	Anterior hypothalamic area posterior part
**AHiAL**	Amygdalohippocampal area anterolateral part
**AHiPL**	Amygdalohippocampal area posterolateral part
**AHiPM**	Amygdalohippocampal area posteromedial part
**AID**	Agranular insular cortex dorsal part
**AIP**	Agranular insular cortex posterior part
**AIV**	Agranular insular cortex ventral part
**AM**	Anteromedial thalamic nucleus
**AmbC**	Ambiguus nucleus compact part
**AmbL**	Ambiguus nucleus loose part
**Ang**	Angular thalamic nucleus
**AOBepl**	External plexiform layer of the accessory olfactory bulb
**AOBgl**	Granule cell layer of the accessory olfactory bulb
**AOBml**	Mitral cell layer of the accessory olfactory bulb
**AON**	Anterior olfactory nucleus
**AP**	Area postrema
**APir**	Amygdalopiriform transition area
**APTD**	Anterior pretectal nucleus dorsal part
**APTV**	Anterior pretectal nucleus ventral part
**ArcD**	Arcuate nucleus dorsal part
**ArcL**	Arcuate nucleus lateral part
**ArcLP**	Arcuate hypothalamic nucleus lateroposterior part
**ArcMP**	Arcuate hypothalamic nucleus medial posterior part
**AStr**	Amygdalostriatal transition area
**ATg**	Anterior tegmental nucleus
**Au1**	Primary auditory cortex
**AuD**	Secondary auditory cortex dorsal area

**AuV**	Secondary auditory cortex ventral area
**AV**	Anteroventral thalamic nucleus
**AVPe**	Anteroventral periventricular nucleus [Anterior hypothalamic area]
**B**	Basal nucleus Meynert
**B9**	B9 serotonin cells
**BAC**	Bed nucleus of the anterior commissure
**BAOT**	Bed nucleus of the accessory olfactory tract
**Bar**	Barringtons nucleus
**BIC**	Nucleus of the brachium of the inferior colliculus
**BLA**	Anterior basolateral nucleus
**BLP**	Posterior basolateral nucleus
**BLV**	Ventral basolateral nucleus
**BMA**	Anterior basomedial nucleus
**BMP**	Posterior basomedial nucleus
**Bo**	Boetzinger complex
**BSTd**	Bed nucleus of the stria terminalis dorsal nucleus
**BSTIA**	Bed nucleus of the stria terminalis intraamygdaloid division
**BSTLD**	Bed nucleus of the stria terminalis lateral division dorsal part
**BSTLI**	Bed nucleus of the stria terminalis lateral division intermedi-ate part
**BSTLJ**	Bed nucleus of the stria terminalis lateral division juxtacapsu-lar part
**BSTLP**	Bed nucleus of the stria terminalis lateral division posterior part
**BSTLV**	Bed nucleus of the stria terminalis lateral division ventral part
**BSTMA**	Bed nucleus of the stria terminalis medial division anterior part
**BSTMAl**	Bed nucleus of the stria terminalis anterior medial part lateral subpart
**BSTMAm**	Bed nucleus of the stria terminalis anterior medial part medial subpart
**BSTMP**	Bed nucleus of the stria terminalis medial division posterior part
**BSTMPI**	Bed nucleus of the stria terminalis medial division postero-intermediate part
**BSTMPL**	Bed nucleus of the stria terminalis medial division posterol-ateral part
**BSTMPM**	Bed nucleus of the stria terminalis medial division postero-medial part
**BSTMV**	Bed nucleus of the stria terminalis medial division ventral part
**BSTSl**	Supracapsular bed nucleus of the stria terminalis lateral part
**BSTSm**	Supracapsular bed nucleus of the stria terminalis medial part
**C1**	C1 adrenaline cells
**C2**	C2 adrenaline cells
**C3**	C3 adrenaline cells
**CA1**	Field CA1 of hippocampus
**CA2**	Field CA2 of hippocampus
**CA3**	Field CA3 of hippocampus
**CeC**	Capsular part
**CeL**	Central amygdaloid nucleus lateral division
**CeM**	Central amygdaloid nucleus medial division
**CEnt**	Caudomedial entorhinal cortex
**CERC**	Cerebellar cortex
**Cg1**	Cingulate cortex area 1
**Cg2**	Cingulate cortex area 2
**CGA**	Central gray alpha part
**CGP**	Central gray pons part
**CI**	Caudal interstitial nucleus of the medial longitudinal fascicu-lus
**CIC**	Central nucleus of the inferior colliculus
**CL**	Centrolateral thalamic nucleus
**CLi**	Caudal linear nucleus of the raphe
**CM**	Central medial thalamic nucleus
**CMAM**	Mammillary body
**CnFD**	Cuneiforme nucleus dorsal part
**CnFV**	Cuneiforme nucleus ventral part
**COAp**	Posterior amygdaloid nucleus
**Com**	Commissural nucleus of the inferior colliculus
**CPu**	Caudate putamen
**CPud**	Dorsal striatum
**CPuim**	Intermediate caudate putamen

**CPul**	Lateral striatum
**Cu**	Cuneate nucleus
**CVL**	Caudoventral reticular nucleus
**CVLMl**	Caudal ventrolateral medulla lateral part
**CxA1**	Cortex amygdala transition zone layer 1
**DA**	Dorsal hypothalamic area
**DCDp**	Dorsal cochlear nucleus deep core
**DCeN**	Cerebellar nuclei
**DCFu**	Dorsal cochlear nucleus fusiform layer
**DCIC**	Dorsal cortex of the inferior colliculus
**DCl**	Dorsal part of claustrum
**DCNsL**	Dorsal cochlear nucleus superficial layer
**DI**	Dysgranular insular cortex
**DIEnt**	Dorsal intermediate entorhinal cortex
**Dk**	Nucleus of Darkschewitsch
**DLEnt**	Dorsolateral entorhinal cortex
**DLG**	Dorsal geniculate nucleus
**DLL**	Dorsal nucleus of the lateral lemniscus
**DLO**	Dorsolateral orbital cortex
**DLPAG**	Dorsolateral periaqueductal gray
**DMC**	Dorsomedial hypothalamic nucleus compact part
**DMD**	Dorsomedial hypothalamic nucleus dorsal part
**DMPAG**	Dorsomedial periaqueductal gray
**DMTg**	Dorsomedial tegmental area
**DMV**	Dorsomedial hypothalamic nucleus ventral part
**DP**	Dorsal peduncular cortex
**DpG**	Deep gray layer of the superior colliculus
**DPGi**	Dorsal paragigantocellular nucleus
**DPO**	Dorsal periolivary region
**DPPn**	Dorsal peduncular pontine nucleus
**DpWh**	Deep white layer of the superior colliculus
**DRC**	Dorsal raphe nucleus caudal part
**DRD**	Dorsal raphe nucleus dorsal part
**DRI**	Dorsal raphe nucleus interfascicular part
**DRlw**	Dorsal raphe nucleus lateral wing
**DRV**	Dorsal raphe nucleus ventral part
**DTgC**	Dorsal tegmental nucleus central part
**DTgP**	Dorsal tegmental nucleus pericentral part
**DTM**	Dorsal tuberomammillary nucleus
**DTr**	Dorsal transition zone
**DTT**	Dorsal tenia tecta
**ECICL1**	External cortex of the inferior colliculus layer 1
**ECICL2**	External cortex of the inferior colliculus layer 2
**ECICL3**	External cortex of the inferior colliculus layer 3
**ECu**	External cuneate nucleus
**EpP**	Epipeduncular nucleus
**ESO**	Episupraoptic nucleus
**Eth**	Ethmoid thalamic nucleus
**EVe**	Nucleus of origin of efferents of the vestibular nerve
**EW**	Edinger Westphal nucleus
**F**	Nucleus of the fields of Forel
**FC**	Fasciola cinereum
**Fl**	Flocculus
**Fr3**	Frontal cortex area 3
**Fu**	Bed nucleus of the stria terminalis fusiform part
**FVe**	F cell group of the vestibular complex
**GeL**	Substantia gelatinosa of the trigeminal sensory nuclear com-plex
**Gem**	Gemini hypothalamic nucleus
**GI**	Granular insular cortex
**GiA**	Gigantocellular reticular nucleus alpha part

**GiV**	Gigantocellular reticular nucleus ventral part
**GlA**	Glomerular layer accessory olfactory bulb
**Gr**	Gracile nucleus principal part
**GrDG**	Granular layer of the dentate gyrus
**HDB**	Nucleus of the horizontal limb of the diagonal band
**I**	Intercalated nuclei of the amygdala
**I8**	Interstitial nucleus of the vestibulocochlear nerve
**IAM**	Interoanteromedial thalamic nucleus
**IcaM**	Intercalated amygdaloid nucleus main part
**ICjM**	Islands of Calleja major island
**ID**	Interstitial nucleus of the decussation of the superior cerebellar peduncle
**IF**	Interfascicular nucleus
**IG**	Indusium griseum
**IGL**	Intergeniculate leaf
**II**	Intermediate interstitial nucleus of the medial longitudinal fasciculus
**IL**	Infralimbic cortex
**ILL**	Intermediate nucleus of the lateral lemniscus
**IMA**	Intramedullary thalamic area
**IMD**	Intermediodorsal thalamic nucleus
**IMG**	Amygdaloid intramedullary gray
**iml**	Internal medullary lamina
**In**	Intercalated nucleus of the medulla
**InC**	Interstitial nucleus of Cajal
**InG**	Intermediate gray layer of the superior colliculus
**InWh**	Intermediate white layer of the superior colliculus
**IOA**	Inferior olive subnucleus A of medial nucleus
**IOB**	Inferior olive subnucleus B of medial nucleus
**IOBe**	Inferior olive beta subnucleus
**IOC**	Inferior olive subnucleus C of medial nucleus
**IOD**	Inferior olive dorsal nucleus
**IODM**	Inferior olive dorsomedial cell group
**IOK**	Inferior olive cap of Kooy of the medial nucleus
**IOVL**	Inferior olive ventrolateral protrusion
**IPA**	Interpeduncular nucleus apical subnucleus
**IPACL**	Interstitial nucleus of the posterior limb of the anterior com-missure lateral part
**IPACM**	Interstitial nucleus of the posterior limb of the anterior com-missure medial part
**IPC**	Interpeduncular nucleus caudal subnucleus
**IPDL**	Interpeduncular nucleus dorsolateral subnucleus
**IPDM**	Interpeduncular nucleus dorsomedial subnucleus
**IPI**	Interpeduncular nucleus intermediate subnucleus
**IPL**	Interpeduncular nucleus lateral subnucleus
**IPR**	Interpeduncular nucleus rostral subnucleus
**IRtA**	Intermediate reticular nucleus alpha part
**IS**	Inferior salivatory nucleus
**isRT**	Isthmic reticular formation
**JPV**	Juxtaparaventricular part [Lateral hypothalamic area [Preoptic anterior region]]
**JxO**	Juxtaolivary nucleus
**KF**	Koelliker Fuse nucleus
**LaDL**	Dorsolateral part of the lateral nucleus
**LAH**	Lateroanterior hypothalamic nucleus
**LaVL**	Ventrolateral part of the lateral nucleus
**LaVM**	Ventromedial part of the lateral nucleus
**LC**	Locus coeruleus
**Ld**	Lambdoid septal zone
**LDDM**	Laterodorsal thalamic nucleus dorsomedial part
**LDTgV**	Laterodorsal tegmental nucleus ventral part
**LDVL**	Laterodorsal thalamic nucleus ventrolateral part
**LEnt**	Lateral entorhinal cortex
**LGP**	Lateral globus pallidus
**LH**	Lateral hypothalamic area

**LHbL**	Lateral habenular nucleus lateral part
**LHbM**	Lateral habenular nucleus medial part
**LHTUC**	Lateral hypothalamic area [Region of tuber cinereum]
**Li**	Linear nucleus of the medulla
**LNol**	Lacunosum molecular layer of the hippocampus
**LO**	Lateral orbital cortex
**LOTL1**	Nucleus of the lateral olfactory tract layer 1
**LOTL2**	Nucleus of the lateral olfactory tract layer 2
**LOTL3**	Nucleus of the lateral olfactory tract layer 3
**LPAG**	Lateral periaqueductal gray
**LPB**	Lateral parabrachial nucleus
**LPBGiA**	Lateral paragigantocellular nucleus alpha part
**LPBGiE**	Lateral paragigantocellular nucleus external part
**LPLC**	Lateral posterior thalamic nucleus laterocaudal part
**LPLR**	Lateral posterior thalamic nucleus laterorostral part
**LPMC**	Lateral posterior thalamic nucleus mediocaudal part
**LPMR**	Lateral posterior thalamic nucleus mediorostral part
**LPO**	Lateral preoptic area
**LRtPC**	Lateral reticular nucleus parvicellular part
**LRtS5**	Lateral reticular nucleus subtrigeminal part
**LSD**	Lateral septal nucleus dorsal part
**LSI**	Lateral septal nucleus intermediate part
**LSO**	Lateral superior olive
**LSS**	Lateral stripe of the striatum
**LSV**	Lateral septal nucleus ventral part
**LTeN**	Lateral terminal nucleus of the accessory optic tract
**LTer**	Lamina terminalis
**LVe**	Lateral vestibular nucleus
**LVPO**	Lateroventral periolivary nucleus
**MA3**	Medial accessory oculomotor nucleus
**MCLH**	Magnocellular nucleus of the lateral hypothalamus
**MCPC**	Magnocellular nucleus of the posterior commissure
**MCPO**	Magnocellular preoptic nucleus
**MDC**	Mediodorsal thalamic nucleus central part
**MDL**	Mediodorsal thalamic nucleus lateral part
**MDM**	Mediodorsal thalamic nucleus medial part
**MdVL**	Medullary reticular nucleus ventrolateral part
**ME**	Median eminence
**Me5**	Mesencephalic trigeminal nucleus
**MeAD**	Medial amygdaloid nucleus anterodorsal part
**MeAV**	Medial amygdaloid nucleus anteroventral part
**MEI**	Medial eminence internal layer
**MEntR**	Medial entorhinal cortex rostral part
**MePD**	Medial amygdaloid nucleus posterodorsal part
**MePV**	Medial amygdaloid nucleus posteroventral part
**MGD**	Medial geniculate nucleus dorsal part
**MGM**	Medial geniculate nucleus medial part
**MGP**	Medial globus pallidus
**MGV**	Medial geniculate nucleus ventral part
**MHb**	Medial habenular nucleus
**MiTg**	Microcellular tegmental nucleus
**MnPO**	Median preoptic nucleus
**MnR**	Median raphe nucleus
**MO**	Medial orbital cortex
**Mo5**	Motor trigeminal nucleus
**Mol**	Molecular layer of the dentate gyrus
**MPA**	Medial preoptic area
**MPL**	Medial paralemniscal nucleus
**MPO**	Medial preoptic nucleus

**MPOC**	Medial preoptic nucleus central part
**MPOL**	Medial preoptic nucleus lateral part
**MPOM**	Medial preoptic nucleus medial part
**MPT**	Medial pretectal nucleus
**MS**	Medial septal nucleus
**MSO**	Medial superior olive
**MT**	Medial terminal nucleus of the accessory optic tract
**MTuN**	Medial tuberal nucleus
**MVe**	Medial vestibular nucleus
**MVPO**	Medioventral periolivary nucleus
**Mx**	Matrix region of the medulla
**MZMG**	Marginal zone of the medial geniculate
**NC**	Nucleus circularis
**NCAT**	Nucleus of the central acoustic tract
**Neck**	Nucleus of the spinal accessory nerve
**Nv**	Navicular nucleus of the basal forebrain
**O**	Nucleus O
**OcxL**	Olfactory cortex layers
**Op**	Optic nerve layer of the superior colliculus
**OPC**	Oval paracentral thalamic nucleus
**OPT**	Olivary pretectal nucleus
**OT**	Nucleus of the optic tract
**OV**	Olfactory ventricle
**p1PAG**	P1 periaqueductal gray
**p1RT**	Reticular thalamic nucleus prosomere 1
**P5**	Peritrigeminal zone
**P7**	Perifacial zone
**Pa4**	Paratrochlear nucleus
**Pa5**	Paratrigeminal nucleus
**Pa6**	Paraabducens nucleus
**PaAMp**	Paraventricular nucleus of the hypothalamus magnocellular division posterior magnocellular part
**PaAP**	Paraventricular hypothalamic nucleus anterior parvicellular part [Periventricular zone anterior region]
**PaDC**	Paraventricular hypothalamic nucleus dorsal cap [Periventricu-lar zone anterior region]
**PaLM**	Paraventricular hypothalamic nucleus lateral magnocellular part [Periventricular zone anterior region]
**PaMP**	Paraventricular hypothalamic nucleus medial parvicellular part [Periventricular zone anterior region]
**PaPo**	Paraventricular hypothalamic nucleus posterior part [Periventricular zone anterior region]
**PaR**	Pararubral nucleus
**PaS**	Parasubiculum
**PaV**	Paraventricular hypothalamic nucleus ventral part [Periventric-ular zone anterior region]
**PBG**	Parabigeminal nucleus
**PBnMe**	Parabrachial nucleus medial
**PBP**	Parabrachial pigmented nucleus
**PC**	Paracentral thalamic nucleus
**PCGS**	Parachochlear glial substance
**PCnFa**	Precuneiform area
**PCom**	Nucleus of the posterior commissure
**PCRtA**	Parvicellular reticular nucleus alpha part
**PDP**	Posterodorsal preoptic nucleus
**PDR**	Posterodorsal raphe nuclei
**PDTg**	Posterodorsal tegmental nucleus
**PeRHO**	Area periventricularis hypothalamica communis [Regio hypothalamus oralis]
**PF**	Parafascicular thalamic nucleus
**PH**	Posterior hypothalamic nucleus

**PHD**	Posterior hypothalamic area dorsal part
**PIL**	Posterior intralaminar thalamic nucleus
**Pir1a**	Piriform cortex layer 1a
**Pir1b**	Piriform cortex layer 1b
**PirL2**	Piriform cortex layer 2
**PirL3**	Piriform cortex layer 3
**PiSt**	Pineal stalk
**PLCo**	Posterolateral cortical nucleus
**PLi**	Posterior limitans thalamic nucleus
**PMCo**	Posteromedial cortical nucleus
**PMn**	Paramedian reticular nucleus
**PMnR**	Paramedian raphe nucleus
**PN**	Paranigral nucleus
**Pn**	Pontine nuclei
**PNC**	Pontine reticular nucleus caudal part
**PnO**	Pontine reticular nucleus oral part
**PnR**	Pontine raphe nucleus
**PnV**	Pontine reticular nucleus ventral part
**Po**	Posterior thalamic nuclear group
**POAd7fn**	Periolivary area between superior olivary nucleus and descend-ing root of the facial nerve
**PoMn**	Posteromedian thalamic nucleus
**POR**	Postrhinal cortex
**Post**	Postsubiculum
**PP**	Peripeduncular nucleus
**PPT**	Posterior pretectal nucleus
**PPTg**	Pedunculopontine tegmental nucleus
**PPy**	Parapyramidal nucleus
**Pr**	Prepositus nucleus
**PR**	Prerubral field
**Pr5**	Principal sensory trigeminal nucleus
**PrBo**	Pre Boetzinger complex
**PrC**	Precommissural nucleus
**PrL**	Prelimbic cortex
**PrS**	Presubiculum
**PS**	Parastriatal nucleus
**PSol**	Parasolitary nucleus
**PT**	Paratenial thalamic nucleus
**PtA**	Parietal association cortex
**PV**	Paraventricular thalamic nucleus
**PVA**	Paraventricular thalamic nucleus anterior part
**PVP**	Paraventricular thalamic nucleus posterior part
**RAmb**	Retroambiguus nucleus
**Rbd**	Rhabdoid nucleus
**RChL**	Retrochiasmatic area lateral part
**Re**	Reuniens thalamic nucleus
**REth**	Retroethmoid nucleus
**Rh**	Rhomboid thalamic nucleus
**RI**	Rostral interstitial nucleus of medial longitudinal fasciculus
**RIP**	Raphe interpositus nucleus
**RL**	Retrolemniscal nucleus
**RLi**	Rostral linear nucleus of the raphe
**RMC**	Red nucleus magnocellular part
**RMg**	Raphe magnus nucleus
**Ro**	Nucleus ofoller
**ROb**	Raphe obscurus nucleus
**ROC**	Red nucleus parvicellular part
**RPa**	Raphe pallidus nucleus
**RPF**	Retroparafascicular nucleus
**RR**	Retrorubral nucleus
**RRE**	Retroreuniens area
**RRF**	A8 dopamine cells retrorubral group

**RSd**	Retrosplenial dorsal
**RSGaL**	Retrosplenial granular a cortex layers
**RSGbL**	Retrosplenial granular b cortex layers
**RSGC**	Retrosplenial granular cortex caudal part
**RSGcC**	Retrosplenial granular cortex c region caudal part
**RtTgL**	Reticulotegmental nucleus of the pons lateral part
**RtTgP**	Reticulotegmental nucleus of the pons pericentral part
**RVL**	Rostroventrolateral reticular nucleus
**RVRG**	Rostral ventral respiratory group
**S**	Subiculum
**S1**	Primary somatosensory cortex
**S2**	Secondary somatosensory cortex
**S5**	Sensory root of the trigeminal nerve
**Sag**	Sagulum nucleus
**SChDL**	Suprachiasmatic nucleus dorsolateral part
**SChVM**	Suprachiasmatic nucleus ventromedial part
**SCO**	Subcommissural organ
**SCzo**	Superior colliculus zonal layer
**SFi**	Septofimbrial nucleus
**SFO**	Subfornical organ
**SG**	Suprageniculate thalamic nucleus
**SGe**	Supragenual nucleus
**SHi**	Septohippocampal nucleus
**SHy**	Septohypothalamic nucleus
**SIB**	Substantia innominata basal part
**SLEAc**	Central division of sublenticular extended amygdala
**SLEAm**	Medial division of the sublenticular extended amygdala
**SM**	Nucleus of the stria medullaris
**SNC**	Substantia nigra compact part
**SNL**	Substantia nigra lateral part
**SNR**	Substantia nigra reticular part
**SolC**	Nucleus of the solitary tract commissural part
**SolCe**	Nucleus of the solitary tract central part
**SolDL**	Nucleus of the solitary tract dorsolateral part
**SolDM**	Nucleus of the solitary tract dorsomedial part
**SolG**	Nucleus of the solitary tract gelatinous part
**Soli**	Nucleus of the solitary tract interstitial part
**SolIM**	Nucleus of the solitary tract intermediate part
**SolL**	Nucleus of the solitary tract lateral part
**SolM**	Nucleus of the solitary tract medial part
**SolRL**	Nucleus of the solitary tract rostrolateral part
**SolV**	Nucleus of the solitary tract ventral part
**SolVL**	Nucleus of the solitary tract ventrolateral part
**SOR**	Supraoptic nucleus retrochiasmatic part
**Sp5nc**	Spinal trigeminal nucleus
**SPa**	Subparaventricular zone of the hypothalamus
**SPFPC**	Submedius thalamic nucleus parvicellular part
**SPFr**	Subparafascicular thalamic nucleus rostral part
**Sph**	Sphenoid nucleus
**SPO**	Superior paraolivary nucleus
**SPTg**	Subpeduncular tegmental nucleus
**SpVe**	Spinal vestibular nucleus
**StA**	Strial part of the preoptic area
**STh**	Subthalamic nucleus
**StHy**	Striohypothalamic nucleus
**Su3**	Supraoculomotor periaqueductal gray
**Su3C**	Supraoculomotor cap
**Su5**	Supratrigeminal nucleus

**SubB**	Subbrachial nucleus
**SubCA**	Subcoeruleus nucleus alpha part
**SubCD**	Subcoeruleus nucleus dorsal part
**SubCV**	Subcoeruleus nucleus ventral part
**SubD**	Submedius thalamic nucleus dorsal part
**SubI**	Subincertal nucleus
**SubP**	Subpostrema area
**SubV**	Submedius thalamic nucleus ventral part
**SuG**	Superficial gray layer of the superior colliculus
**SuS**	Superior salivatory nucleus
**SuVe**	Superior vestibular nucleus
**TC**	Tuber cinereum area
**Te**	Terete hypothalamic nucleus
**TeA**	Temporal association cortex 1
**TS**	Triangular septal nucleus
**TuOd**	Olfactory tubercle densocellular layer
**TuOLa1**	Olfactory tubercle plexiform layer
**TuOpo**	Olfactory tubercle polymorph layer
**Tz**	Nucleus of the trapezoid body
**V1**	Primary visual cortex
**V1B**	Primary visual cortex binocular area
**V1M**	Primary visual cortex monocular area
**V2L**	Secondary visual cortex lateral area
**V2ML**	Secondary visual cortex mediolateral area
**V2MM**	Secondary visual cortex mediomedial area
**VA**	Ventro anterior thalamic nucleus
**VCA**	Ventral cochlear nucleus anterior part
**VCAGr**	Ventral cochlear nucleus granule cell layer
**VCl**	Ventral part of claustrum
**VCP**	Ventral cochlear nucleus posterior part
**VDB**	Nucleus of the vertical limb of the diagonal band
**VeCb**	Vestibulocerebellar nucleus
**VEn**	Ventral endopiriform nucleus
**VG**	Ventral geniculate nucleus
**VIEnt**	Ventral intermediate entorhinal cortex
**VL**	Ventrolateral thalamic nucleus
**VLG**	Ventral lateral geniculate nucleus
**VLH**	Ventrolateral hypothalamic nucleus
**VLL**	Ventral nucleus of the lateral lemniscus
**VLPAG**	Ventrolateral periaqueductal gray
**VLPO**	Ventrolateral preoptic nucleus
**VM**	Ventromedial thalamic nucleus
**VMHC**	Ventromedial hypothalamic nucleus central part
**VMHDM**	Ventromedial hypothalamic nucleus dorsomedial part
**VMHVL**	Ventromedial hypothalamic nucleus ventrolateral part
**VMPO**	Ventromedial preoptic nucleus
**VO**	Ventral orbital cortex
**VOLT**	Vascular organ of the lamina terminalis
**VP**	Ventral pallidum
**VPL**	Ventral posterolateral thalamic nucleus
**VPM**	Ventral posteromedial thalamic nucleus
**VPPC**	Ventral posterior thalamic nucleus parvicellular part
**VRe**	Ventral reuniens thalamic nucleus
**VTAR**	Ventral tegmental area rostral part
**VTg**	Ventral tegmental nucleus
**VTM**	Ventral tuberomammillary nucleus
**VTT**	Ventral tenia tecta
**X**	Nucleus X
**Xi**	Xiphoid thalamic nucleus

**Y**	Nucleus Y
**Z**	Nucleus Z
**ZIC**	Zona incerta caudal part
**ZID**	Zona incerta dorsal part
**ZIR**	Zona incerta rostral part
**ZIV**	Zona incerta ventral part
**ZL**	Zona limitans
