# Supplementary material for: Connectome-based prediction of functional impairment in experimental stroke models
Source: PLoS One. 2024 Dec 19;19(12):e0310743. doi: 10.1371/journal.pone.0310743 (PMC11658581; doi:10.1371/journal.pone.0310743)
Supplement: S2 Table — The column “Region” contains non-lesioned regions and the column “Max” the number of connections of non-lesioned regions with lesioned regions. The regions with functional markers (F) are motor (M) or learning regions (L). E.g., the medial agranular prefrontal cortex is a motor region (M) and has 15 connections with ICH lesioned regions. In the last column of non-lesioned regions which have only 1 connection with ICH lesioned region no functional assignments are available. Anterodorsal thalamic nucleus rostral part (L), anteroventral thalamic nucleus ventral part (L), field CA2 of hippocampus (L), presubiculum (L), cerebellar cortex (M), cerebellar nuclei (M) and pontine nuclei (M) are non-lesioned regions with functional assignments, however, without connections from or to ICH lesioned regions. (PDF) [file pone.0310743.s008.pdf]

S1 Table. Sorted sum of connections of non-lesioned and lesioned ICH regions. The column “Region” contains non-lesioned regions and the column “Max” the number of connections of non-lesioned regions with lesioned regions. The regions with functional markers (F) are motor (M) or learning regions (L). E.g., the medial agranular prefrontal cortex is a motor region (M) and has 15 connections with ICH lesioned regions. In the last column of non-lesioned regions which have only 1 connection with ICH lesioned region no functional assignments are available. Anterodorsal thalamic nucleus rostral part (L), anteroventral thalamic nucleus ventral part (L), field CA2 of hippocampus (L), presubiculum (L), cerebellar cortex (M), cerebellar nuclei (M) and pontine nuclei (M) are non-lesioned regions with functional assignments, however, without connections from or to ICH lesioned regions.

Region	Max	F	Region	Max	F	Region	Max	F	Region	Max
Lateral hypothalamic area	16		Medial globus pallidus	5	M	Medial amygdaloid nucleus anteroventral part	3		Red nucleus parvicellular part	1
Medial agranular prefrontal cortex	15	M	Flocculus	5		Bed nucleus of the accessory olfactory tract	3		Interfascicular nucleus	1
Prelimbic cortex	15		Pontine reticular nucleus oral part	5		Accumbens nucleus core	3		Paranigral nucleus	1
Infralimbic cortex	14		Koelliker Fuse nucleus	5		Ventral intermediate entorhinal cortex	3		Dorsomedial tegmental area	1
Agranular insular cortex dorsal part	14		Principal sensory trigeminal nucleus	5		Anterior olfactory nucleus	3		Perifacial zone	1
Locus coeruleus	12		Lateral vestibular nucleus	5		Interoanteromedial thalamic nucleus	2	L	Peritrigeminal zone	1
Ventral pallidum	12		Dorsal motor nucleus of vagus	5		Rhomboid nucleus	2	L	Nucleus of the solitary tract central part	1
Dysgranular insular cortex	12		Rostral linear nucleus of the raphe	5		Subparafascicular thalamic nucleus rostral part	2	L	Nucleus of the solitary tract gelatinous part	1
Substantia nigra compact part	11	M	Rhomboid thalamic nucleus	5		Ventral tegmental area rostral part	2		Nucleus of the solitary tract rostrolateral part	1
Substantia nigra reticular part	11	M	Reuniens thalamic nucleus	5		Ventral tegmental nucleus	2		Dorsal raphe nucleus lateral wing	1
Pedunculopontine tegmental nucleus	11		Paraventricular thalamic nucleus anterior part	5		A7 noradrenaline cells	2		Paramedian raphe nucleus	1
Primary somatosensory cortex	11		Paraventricular thalamic nucleus posterior part	5		Barringtons nucleus	2		Raphe interpositus nucleus	1
Secondary somatosensory cortex	11		Posterior intralaminar thalamic nucleus	5		Gigantocellular reticular nucleus alpha part	2		Raphe pallidus nucleus	1
Perirhinal cortex	10	L	Bed nucleus of the stria terminalis fusiform part	5		Parvicellular reticular nucleus alpha part	2		Caudal ventrolateral medulla lateral part	1
Lateral agranular prefrontal cortex	10	M	Supracapsular bed nucleus of the stria terminalis lateral part	5		C2 adrenaline cells	2		A1 noradrenergic cells	1
Substantia nigra lateral part	10		Central division of sublenticular extended amygdala	5		C3 adrenaline cells	2		Caudoventral reticular nucleus	1
Parabrachial nucleus medial	10		Posteromedial cortical nucleus	5		Area postrema	2		Gracile nucleus principal part	1
A8 dopamine cells retrorubral group	10		Intercalated nuclei of the amygdala	5		Spinal trigeminal nucleus	2		Superior salivatory nucleus	1
Posterior basolateral nucleus	10		Ectorhinal cortex	5		Nucleus of the solitary tract commissural part	2		Nucleus of the vertical limb of the diagonal band	1
Agranular insular cortex posterior part	10		Dorsal peduncular cortex	5		Nucleus of the solitary tract dorsolateral part	2		Median preoptic nucleus	1
Granular insular cortex	10		Secondary visual cortex lateral area	5		Nucleus of the solitary tract dorsomedial part	2		Medial preoptic area	1
Lateral orbital cortex	10		Lateral entorhinal cortex	4	L	Nucleus of the solitary tract intermediate part	2		Ventral tenia tecta	1
Caudate putamen	9	M	Lateral globus pallidus	4	M	Nucleus of the solitary tract medial part	2		Paraventricular hypothalamic nucleus ventral part	1
Central medial thalamic nucleus	9		Subthalamic nucleus	4	M	Nucleus of the solitary tract ventral part	2		Retrochiasmatic area lateral part	1
Paraventricular thalamic nucleus	9		Lateral periaqueductal gray	4		Dorsal raphe nucleus dorsal part	2		Retroethmoid nucleus	1
Anterior basomedial nucleus	9		Parabrachial pigmented nucleus	4		Dorsal raphe nucleus ventral part	2		Retroparafascicular nucleus	1
Amygdalopiriform transition area	9		Edinger Westphal nucleus	4		Pontine raphe nucleus	2		Mediodorsal thalamic nucleus central part	1
Medial orbital cortex	9		Spinal vestibular nucleus	4		Medial septal nucleus	2		Subfornical organ	1
Caudal linear nucleus of the raphe	8		Medial vestibular nucleus	4		Substantia innominata basal part	2		Ventral posterior thalamic nucleus parvicellular part	1
Lateral preoptic area	8		Raphe magnus nucleus	4		Magnocellular preoptic nucleus	2		Ethmoid thalamic nucleus	1
Parafascicular thalamic nucleus	8		Ventromedial hypothalamic nucleus central part	4		Dorsal tenia tecta	2		Laterodorsal thalamic nucleus dorsomedial part	1
Paratenial thalamic nucleus	8		Suprageniculate thalamic nucleus	4		Lateroanterior hypothalamic nucleus	2		Nucleus of the stria medullaris	1
Agranular insular cortex ventral part	8		Bed nucleus of the stria terminalis intraamygdaloid division	4		Ventromedial hypothalamic nucleus dorsomedial part	2		Posteromedian thalamic nucleus	1
Subiculum	7	L	Bed nucleus of the stria terminalis lateral division posterior part	4		Paraventricular hypothalamic nucleus lateral magnocellular part	2		Nucleus of the fields of Forel	1
Ventrolateral periaqueductal gray	7		Bed nucleus of the stria terminalis medial division anterior part	4		Supraoptic nucleus retrochiasmatic part	2		Zona incerta caudal part	1
Retrorubral nucleus	7		Dentate gyrus	3	L	Anterior hypothalamic area central part	2		Zona incerta ventral part	1
Pontine reticular nucleus caudal part	7		Parasubiculum	3	L	Posterior limitans thalamic nucleus	2		Lateral habenular nucleus lateral part	1
Lateral parabrachial nucleus	7		Cingulate cortex area 1	3	L	Posterior thalamic nuclear group triangular part	2		Medial habenular nucleus	1
Median raphe nucleus	7		Cingulate cortex area 2	3	L	Intermediodorsal thalamic nucleus	2		Bed nucleus of the stria terminalis medial division posterointermediate part	1
Cuneate nucleus	7		Central gray pons part	3		Mediodorsal thalamic nucleus medial part	2		Bed nucleus of the stria terminalis dorsal nucleus	1
Posterior basomedial nucleus	7		Nucleus of Darkschewitsch	3		Zona incerta dorsal part	2		Nucleus of the lateral olfactory tract layer 1	1
Anterior amygdaloid area	7		Subpeduncular tegmental nucleus	3		Ventrolateral part of the lateral nucleus	2		Nucleus of the lateral olfactory tract layer 3	1
Anterior cortical amygdaloid nucleus	7		Interstitial nucleus of Cajal	3		Ventromedial part of the lateral nucleus	2		Amygdalohippocampal area anterolateral part	1
Medial amygdaloid nucleus anterodorsal part	7		A5 noradrenaline cells	3		Bed nucleus of the stria terminalis lateral division juxtacapsular part	2		Amygdalohippocampal area posteromedial part	1
Frontal cortex area 3	7		Prepositus nucleus	3		Bed nucleus of the stria terminalis medial division posterior part	2		Lateral accumbens shell	1
Primary visual cortex	7		Subcoeruleus nucleus dorsal part	3		Bed nucleus of the stria terminalis medial division ventral part	2		Dorsal part of claustrum	1
Ventral orbital cortex	7		Subcoeruleus nucleus ventral part	3		Medial amygdaloid nucleus posterodorsal part	2		Septohippocampal nucleus	1
Field CA1 of hippocampus	6	L	Paratrigeminal nucleus	3		Ventral basolateral nucleus	2		Postsubiculum	1
Ventrolateral thalamic nucleus	6	M	Supratrigeminal nucleus	3		Dorsolateral entorhinal cortex	2		Dorsal intermediate entorhinal cortex	1
Dorsolateral periaqueductal gray	6		Mesencephalic trigeminal nucleus	3		Dorsolateral orbital cortex	2		Piriform cortex layer 3	1
Peripeduncular nucleus	6		Nucleus of the solitary tract ventrolateral part	3		Medial geniculate nucleus dorsal part	2		Retrosplenial dorsal	1
Superior vestibular nucleus	6		Dorsal raphe nucleus caudal part	3		Medial geniculate nucleus medial part	2		Temporal association cortex 1	1
Paraventricular hypothalamic nucleus anterior parvicellular part	6		A2 noradrenergic cells	3		Medial geniculate nucleus ventral part	2		Primary auditory cortex	1
Dorsolateral part of the lateral nucleus	6		Dorsal paragigantocellular nucleus	3		Mammillary body	1	L	Precommissural nucleus	1
Bed nucleus of the stria terminalis lateral division dorsal part	6		Nucleus of the horizontal limb of the diagonal band	3		Field CA3 of hippocampus	1	L	Ventral lateral geniculate nucleus	1
Bed nucleus of the stria terminalis lateral division ventral part	6		Dorsal hypothalamic area	3		Postrhinal cortex	1	L	Interpeduncular nucleus apical subnucleus	1
Posterior amygdaloid nucleus	6		Parastriatal nucleus	3		Central gray alpha part	1		Interpeduncular nucleus caudal subnucleus	1
Posterolateral cortical nucleus	6		Posterior hypothalamic nucleus	3		Parabigeminal nucleus	1		Interpeduncular nucleus intermediate subnucleus	1
Medial amygdaloid nucleus posteroventral part	6		Ventromedial hypothalamic nucleus ventrolateral part	3		Dorsomedial periaqueductal gray	1		Interpeduncular nucleus rostral subnucleus	1
Parietal association cortex	6		Nucleus of the lateral olfactory tract layer 2	3		Red nucleus magnocellular part	1		Lateral septal nucleus ventral part	1
