# Supplementary material for: Connectome-based prediction of functional impairment in experimental stroke models
Source: PLoS One. 2024 Dec 19;19(12):e0310743. doi: 10.1371/journal.pone.0310743 (PMC11658581; doi:10.1371/journal.pone.0310743)
Supplement: S3 Table — The relation of functional groups (Marker: motor behavior (2), learning behavior (3)) to 6 lesioned regions in the dMCAO model. Regions were sorted by their sum of connections and reciprocal connections. E.g. the lateral enthorinal cortex has 6 connections to all lesioned regions and all of these 6 connections are reciprocal. (PDF) [file pone.0310743.s009.pdf]

S2 Table. Connections of dMCAO lesioned regions to motor and learning regions. The relation of functional groups (Marker: motor behavior (2), learning behavior (3)) to 6 lesioned regions in the dMCAO model. Regions were sorted by their sum of connections and reciprocal connections. E.g. the lateral enthorinal cortex has 6 connections to all lesioned regions and all of these 6 connections are reciprocal.

Region	Sum	PtA	AID	AIV	DI	GI	S1	Reciprocal
Lateral entorhinal cortex	6	1	1	1	1	1	1	6
Perirhinal cortex	6	1	1	1	1	1	1	6
Postrhinal cortex	6	1	1	1	1	1	1	5
Reuniens thalamic nucleus	6	1	1	1	1	1	1	4
Rhomboid thalamic nucleus	6	1	1	1	1	1	1	3
Field CA1 of hippocampus	5	1	1	1	1	1	0	0
Posterior thalamic nuclear group	4	1	0	0	1	1	1	3
Anteroventral thalamic nucleus	4	1	1	0	1	0	1	1
Mammillary body	4	1	1	0	0	1	1	0
Cingulate cortex area 1	3	1	1	0	0	0	1	2
Cingulate cortex area 2	3	1	1	0	0	0	1	2
Presubiculum	3	1	0	1	0	1	0	1
Parasubiculum	3	1	0	1	0	1	0	1
Subiculum	3	1	1	1	0	0	0	0
Anterodorsal thalamic nucleus	1	0	1	0	0	0	0	0
Interoanteromedial thalamic nucleus	1	0	0	0	1	0	0	0
Field CA3 of hippocampus	1	1	0	0	0	0	0	0
Subparafascicular thalamic nucleus rostral part	0	0	0	0	0	0	0	0
Dentate gyrus	0	0	0	0	0	0	0	0
Field CA2 of hippocampus	0	0	0	0	0	0	0	0
Lateral agranular prefrontal cortex	6	1	1	1	1	1	1	6
Medial agranular prefrontal cortex	6	1	1	1	1	1	1	5
Subthalamic nucleus	6	1	1	1	1	1	1	3
Caudate putamen	6	1	1	1	1	1	1	2
Substantia nigra compact part	4	1	0	0	1	1	1	3
Pontine nuclei	4	1	0	1	0	1	1	1
Substantia nigra reticular part	4	1	1	0	0	1	1	0
Ventrolateral thalamic nucleus	3	1	0	1	0	0	1	2
Medial globus pallidus	2	1	0	0	0	0	1	1
Cerebellar cortex	2	1	0	0	0	0	1	0
Cerebellar nuclei	1	0	0	0	0	0	1	0
Lateral globus pallidus	0	0	0	0	0	0	0	0
