# Supplementary material for: Connectome-based prediction of functional impairment in experimental stroke models
Source: PLoS One. 2024 Dec 19;19(12):e0310743. doi: 10.1371/journal.pone.0310743 (PMC11658581; doi:10.1371/journal.pone.0310743)
Supplement: S4 Table — Connections of dMCAO lesioned regions, functionally defined regions and control regions without functional definitions. (PDF) [file pone.0310743.s010.pdf]

**S3 Table. Overview of all connections of dMCAO lesioned regions**. Connections of dMCAO lesioned regions, functionally defined regions and control regions without functional definitions.

| Region | Σ | Reci | Marker | Region | Σ | Reci |
|---|---|---|---|---|---|---|
| Mammillary body | 257 | 77 | Learn | Magnocellular preoptic nucleus | 93 | 19 |
| Perirhinal cortex | 208 | 68 | Learn | Prepositus nucleus | 92 | 24 |
| Subiculum | 191 | 50 | Learn | Basal nucleus Meynert | 92 | 15 |
| Field CA1 of hippocampus | 180 | 45 | Learn | Secondary visual cortex lateral area | 91 | 19 |
| Lateral entorhinal cortex | 173 | 62 | Learn | Paraventricular hypothalamic nucleus anterior parvicellular part | 90 | 12 |
| Rhomboid thalamic nucleus | 138 | 22 | Learn | Accumbens nucleus core | 90 | 23 |
| Posterior thalamic nuclear group | 118 | 17 | Learn | Raphe obscurus nucleus | 89 | 20 |
| Cingulate cortex area 1 | 101 | 29 | Learn | Paraventricular thalamic nucleus posterior part | 89 | 12 |
| Dentate gyrus | 86 | 20 | Learn | Medial amygdaloid nucleus anterodorsal part | 89 | 17 |
| Field CA3 of hippocampus | 83 | 16 | Learn | Posterior intralaminar thalamic nucleus | 88 | 16 |
| Cingulate cortex area 2 | 82 | 20 | Learn | Anterior olfactory nucleus | 88 | 20 |
| Presubiculum | 76 | 22 | Learn | Dorsal peduncular cortex | 84 | 12 |
| Parasubiculum | 75 | 18 | Learn | Rostral linear nucleus of the raphe | 83 | 6 |
| Subparafascicular thalamic nucleus rostral part | 65 | 17 | Learn | Lateral septal nucleus ventral part | 83 | 20 |
| Postrhinal cortex | 65 | 27 | Learn | Ventral tegmental nucleus | 82 | 16 |
| Field CA2 of hippocampus | 51 | 10 | Learn | Retrorubral nucleus | 82 | 17 |
| Interoanteromedial thalamic nucleus | 46 | 8 | Learn | Nucleus of the solitary tract medial part | 81 | 16 |
| Lateral agranular prefrontal cortex | 199 | 58 | Mot | Dorsal striatum | 81 | 15 |
| Medial agranular prefrontal cortex | 190 | 61 | Mot | Nucleus of Darkschewitsch | 80 | 5 |
| Caudate putamen | 177 | 37 | Mot | Ventromedial hypothalamic nucleus central part | 79 | 14 |
| Substantia nigra compact part | 162 | 38 | Mot | Interstitial nc. of the post. limb of the anterior commissure medial part | 79 | 13 |
| Subthalamic nucleus | 157 | 33 | Mot | Capsular part | 78 | 7 |
| Cerebellar nuclei | 126 | 28 | Mot | Rostral ventral respiratory group | 77 | 18 |
| Substantia nigra reticular part | 113 | 23 | Mot | Nucleus of the solitary tract commissural part | 76 | 14 |
| Cerebellar cortex | 106 | 8 | Mot | Mediodorsal thalamic nucleus medial part | 76 | 17 |
| Ventrolateral thalamic nucleus | 83 | 18 | Mot | Paraventricular thalamic nucleus anterior part | 75 | 5 |
| Medial globus pallidus | 75 | 18 | Mot | Lateral vestibular nucleus | 75 | 14 |
| Pontine nuclei | 66 | 10 | Mot | A5 noradrenaline cells | 75 | 12 |
| Lateral globus pallidus | 36 | 7 | Mot | Ventral posteromedial thalamic nucleus | 74 | 16 |
| Lateral hypothalamic area | 485 | 164 | | Superior vestibular nucleus | 74 | 14 |
| Locus coeruleus | 301 | 81 | | Dorsal raphe nucleus lateral wing | 74 | 11 |
| Infralimbic cortex | 288 | 93 | | Dorsal endopiriform nucleus | 74 | 22 |
| Median raphe nucleus | 265 | 73 | | Barringtons nucleus | 74 | 11 |
| Prelimbic cortex | 253 | 80 | | Primary auditory cortex | 73 | 19 |
| Medial preoptic area | 237 | 66 | | Medial amygdaloid nucleus posteroventral part | 73 | 13 |
| Reuniens thalamic nucleus | 219 | 53 | | Intermediodorsal thalamic nucleus | 73 | 9 |
| Ventrolateral periaqueductal gray | 209 | 52 | | Ventromedial hypothalamic nucleus dorsomedial part | 71 | 12 |
| Lateral parabrachial nucleus | 200 | 54 | | Ventral posterolateral thalamic nucleus | 71 | 10 |
| Pedunculopontine tegmental nucleus | 193 | 51 | | Temporal association cortex 1 | 71 | 15 |
| Paraventricular thalamic nucleus | 191 | 49 | | Bed nucleus of the stria terminalis lateral division ventral part | 71 | 10 |
| Parafascicular thalamic nucleus | 183 | 48 | | Anteroventral thalamic nucleus | 70 | 20 |
| Posterior hypothalamic nucleus | 181 | 41 | | Ventral lateral geniculate nucleus | 69 | 16 |
| Lateral preoptic area | 179 | 40 | | Pontine raphe nucleus | 68 | 7 |
| Edinger Westphal nucleus | 178 | 36 | | Lateral septal nucleus intermediate part | 68 | 13 |
| Primary somatosensory cortex | 177 | 51 | | Peripeduncular nucleus | 67 | 9 |
| Raphe magnus nucleus | 176 | 42 | | Medial amygdaloid nucleus posterodorsal part | 65 | 17 |

| Region | Σ | Reci | Marker | Region | Σ | Reci |
|---|---|---|---|---|---|---|
| Central amygdaloid nucleus medial division | 169 | 31 | | Flocculus | 65 | 9 |
| Spinal trigeminal nucleus | 164 | 37 | | Paraventricular hypothalamic nucleus medial parvicellular part | 64 | 9 |
| Dorsomedial hypothalamic nucleus dorsal part | 162 | 1 | | Cuneate nucleus | 64 | 3 |
| Nucleus of the horizontal limb of the diagonal band | 161 | 44 | | Amygdalostriatal transition area | 64 | 7 |
| Parietal association cortex | 160 | 45 | | Spinal vestibular nucleus | 63 | 8 |
| Medial preoptic nucleus | 160 | 44 | | Caudal linear nucleus of the raphe | 62 | 6 |
| Parabrachial nucleus medial | 159 | 36 | | Suprageniculate thalamic nucleus | 61 | 10 |
| Ventral pallidum | 157 | 46 | | Motor trigeminal nucleus | 61 | 6 |
| Medial septal nucleus | 156 | 43 | | Gigantocellular reticular nucleus alpha part | 61 | 9 |
| Central medial thalamic nucleus | 150 | 28 | | Medial habenular nucleus | 60 | 11 |
| Anterior cortical amygdaloid nucleus | 148 | 40 | | Mediodorsal thalamic nucleus lateral part | 59 | 10 |
| Anterior amygdaloid area | 143 | 32 | | Medial geniculate nucleus medial part | 59 | 12 |
| Agranular insular cortex posterior part | 143 | 38 | | Supratrigeminal nucleus | 58 | 5 |
| Central amygdaloid nucleus lateral division | 142 | 23 | | Nucleus of the fields of Forel | 58 | 8 |
| Anterior basomedial nucleus | 134 | 34 | | Substantia nigra lateral part | 57 | 10 |
| Medial orbital cortex | 133 | 36 | | Precommissural nucleus | 57 | 12 |
| Posterior basomedial nucleus | 130 | 30 | | Mediodorsal thalamic nucleus central part | 57 | 10 |
| Nucleus of the vertical limb of the diagonal band | 130 | 40 | | Bed nucleus of the stria terminalis intraamygdaloid division | 57 | 6 |
| Posterior basolateral nucleus | 126 | 35 | | Subfornical organ | 56 | 15 |
| Amygdalopiriform transition area | 126 | 36 | | Dorsolateral entorhinal cortex | 56 | 11 |
| Lateral orbital cortex | 125 | 28 | | Dorsal paragigantocellular nucleus | 56 | 4 |
| Agranular insular cortex dorsal part | 125 | 28 | | Area postrema | 56 | 9 |
| Pontine reticular nucleus oral part | 124 | 25 | | Ventromedial part of the lateral nucleus | 55 | 8 |
| Granular insular cortex | 124 | 31 | | Dorsal raphe nucleus dorsal part | 55 | 0 |
| Dysgranular insular cortex | 124 | 27 | | Ventrolateral preoptic nucleus | 54 | 12 |
| Paratenial thalamic nucleus | 123 | 29 | | Interstitial nucleus of Cajal | 54 | 3 |
| Medial vestibular nucleus | 121 | 26 | | Anteroventral periventricular nucleus [Anterior hypothalamic area] | 54 | 9 |
| Dorsolateral periaqueductal gray | 121 | 13 | | Interfascicular nucleus | 53 | 7 |
| Ectorhinal cortex | 120 | 42 | | Subparaventricular zone of the hypothalamus | 52 | 8 |
| Ventral orbital cortex | 119 | 35 | | Dorsolateral part of the lateral nucleus | 52 | 2 |
| Ventromedial thalamic nucleus | 118 | 21 | | Bed nucleus of the stria terminalis lateral division dorsal part | 52 | 8 |
| Primary visual cortex | 117 | 33 | | Bed nucleus of the stria terminalis dorsal nucleus | 52 | 8 |
| Mesencephalic trigeminal nucleus | 116 | 17 | | Medullary reticular nucleus ventrolateral part | 51 | 4 |
| A8 dopamine cells retrorubral group | 116 | 18 | | Ventrolateral part of the lateral nucleus | 49 | 6 |
| Lateral periaqueductal gray | 114 | 20 | | Ventral basolateral nucleus | 49 | 7 |
| Juxtaparaventricular part [Lateral hypothalamic area] | 114 | 34 | | Retrosplenial dorsal | 49 | 16 |
| Agranular insular cortex ventral part | 114 | 35 | | Lateral accumbens shell | 49 | 3 |
| Pontine reticular nucleus caudal part | 113 | 23 | | Median eminence | 48 | 7 |
| Principal sensory trigeminal nucleus | 112 | 25 | | Ventral tuberomammillary nucleus | 47 | 4 |
| Centrolateral thalamic nucleus | 107 | 16 | | Septofimbrial nucleus | 47 | 8 |
| Posterolateral cortical nucleus | 105 | 31 | | Dorsal raphe nucleus ventral part | 47 | 3 |
| Secondary somatosensory cortex | 104 | 28 | | Anterodorsal thalamic nucleus | 47 | 11 |
| Dorsal raphe nucleus caudal part | 104 | 13 | | Lateral septal nucleus dorsal part | 46 | 6 |
| Dorsal motor nucleus of vagus | 104 | 8 | | Interpeduncular nucleus rostral subnucleus | 46 | 7 |
| Koelliker Fuse nucleus | 103 | 18 | | Bed nucleus of the stria terminalis fusiform part | 46 | 7 |
| Ventromedial hypothalamic nucleus ventrolateral part | 101 | 15 | | Anterior hypothalamic area posterior part | 46 | 10 |
| Dorsal hypothalamic area | 101 | 17 | | Subcoeruleus nucleus dorsal part | 45 | 2 |

| Region | Σ | Reci | Marker | Region | Σ | Reci |
|---|---|---|---|---|---|---|
| Raphe pallidus nucleus | 100 | 17 | | Lateral habenular nucleus medial part | 45 | 7 |
| Anterior basolateral nucleus | 100 | 24 | | Triangular septal nucleus | 44 | 11 |
| Posterior amygdaloid nucleus | 99 | 34 | | Medial amygdaloid nucleus anteroventral part | 44 | 6 |
| Median preoptic nucleus | 96 | 22 | | Anterior hypothalamic area central part | 44 | 6 |
| Posteromedial cortical nucleus | 93 | 25 | | Vascular organ of the lamina terminalis | 43 | 9 |
| Paracentral thalamic nucleus | 93 | 10 | | Nucleus of the solitary tract ventrolateral part | 43 | 9 |
| Zona incerta ventral part | 42 | 4 | | Interpeduncular nucleus intermediate subnucleus | 18 | 3 |
| Ventral tegmental area rostral part | 42 | 2 | | C3 adrenaline cells | 18 | 2 |
| Ventral endopiriform nucleus | 42 | 8 | | Bed nucleus of the stria terminalis medial division posterolateral part | 18 | 1 |
| Postsubiculum | 42 | 14 | | Abducens nucleus | 18 | 1 |
| Intergeniculate leaf | 42 | 4 | | Subincertal nucleus | 17 | 0 |
| Parabrachial pigmented nucleus | 41 | 0 | | Pre Boetzinger complex | 17 | 0 |
| Nucleus of the solitary tract intermediate part | 41 | 4 | | Pontine reticular nucleus ventral part | 17 | 0 |
| Dorsomedial periaqueductal gray | 41 | 6 | | Paraventricular hypoth. nc. lateral magnocellular part | 17 | 0 |
| Rostroventrolateral reticular nucleus | 40 | 4 | | Nucleus of the solitary tract central part | 17 | 1 |
| Central gray pons part | 40 | 3 | | Dorsal tuberomammillary nucleus | 17 | 1 |
| Bed nucleus of the stria terminalis lateral division posterior part | 40 | 4 | | C2 adrenaline cells | 17 | 0 |
| Medial terminal nucleus of the accessory optic tract | 39 | 7 | | B9 serotonin cells | 17 | 0 |
| Medial paralemniscal nucleus | 39 | 3 | | Retroambiguus nucleus | 16 | 2 |
| Central nucleus of the inferior colliculus | 39 | 12 | | Red nucleus parvicellular part | 16 | 1 |
| Central division of sublenticular extended amygdala | 39 | 6 | | Red nucleus magnocellular part | 16 | 2 |
| Medial preoptic nucleus lateral part | 38 | 5 | | Raphe interpositus nucleus | 16 | 0 |
| Dorsolateral orbital cortex | 38 | 10 | | Lateral posterior thalamic nucleus laterorostral part | 16 | 3 |
| Zona incerta dorsal part | 37 | 7 | | Interstitial nc. of the post. limb of the anterior commissure lat. part | 16 | 1 |
| Frontal cortex area 3 | 36 | 5 | | External cuneate nucleus | 16 | 0 |
| Principal hypoglossal nucleus | 35 | 2 | | Caudoventral reticular nucleus | 16 | 0 |
| Paramedian raphe nucleus | 35 | 6 | | BST medial division posterointermediate part | 16 | 1 |
| Dorsal tenia tecta | 35 | 7 | | Subcoeruleus nucleus ventral part | 15 | 0 |
| C1 adrenaline cells | 35 | 2 | | Prerubral field | 15 | 2 |
| Intermediate gray layer of the superior colliculus | 34 | 2 | | Posterior hypothalamic area dorsal part | 15 | 1 |
| Gigantocellular reticular nucleus ventral part | 34 | 4 | | Nucleus of the solitary tract rostrolateral part | 15 | 4 |
| Dorsal intermediate entorhinal cortex | 34 | 6 | | Inferior olive ventrolateral protrusion | 15 | 1 |
| Intercalated nuclei of the amygdala | 33 | 5 | | Inferior olive beta subnucleus | 15 | 1 |
| Dorsal geniculate nucleus | 33 | 6 | | Bed nucleus of the stria terminalis medial division posteromedial part | 15 | 1 |
| Dorsal cortex of the inferior colliculus | 33 | 5 | | Anterior hypothalamic area anterior part | 15 | 2 |
| A2 noradrenergic cells | 33 | 3 | | Trochlear nucleus | 14 | 0 |
| Ventral tenia tecta | 32 | 9 | | Retrosplenial granular cortex c region caudal part | 14 | 3 |
| Nucleus of the solitary tract lateral part | 32 | 4 | | Peritrigeminal zone | 14 | 1 |
| Lateral striatum | 32 | 3 | | Olfactory tubercle polymorph layer | 14 | 0 |
| Lateral habenular nucleus lateral part | 32 | 4 | | Medial accessory oculomotor nucleus | 14 | 2 |
| Deep gray layer of the superior colliculus | 32 | 6 | | Lateral terminal nucleus of the accessory optic tract | 14 | 2 |
| Bed nucleus of the accessory olfactory tract | 32 | 7 | | Intermediate white layer of the superior colliculus | 14 | 1 |
| Paranigral nucleus | 31 | 5 | | Intermediate reticular nucleus alpha part | 14 | 0 |
| Bed nucleus of the stria terminalis medial division ventral part | 31 | 1 | | Intermediate caudate putamen | 14 | 1 |
| A7 noradrenaline cells | 31 | 1 | | Indusium griseum | 14 | 1 |
| Olivary pretectal nucleus | 30 | 4 | | Secondary auditory cortex dorsal area | 13 | 1 |
| Ventral posterior thalamic nucleus parvicellular part | 29 | 3 | | Posterior limitans thalamic nucleus | 13 | 0 |

| Region | Σ | Reci | Marker | Region | Σ | Reci |
|---|---|---|---|---|---|---|
| Olfactory cortex layers | 29 | 5 | | Nucleus of the solitary tract dorsomedial part | 13 | 1 |
| Nucleus of the lateral olfactory tract layer 2 | 29 | 3 | | Lateral reticular nucleus parvicellular part | 13 | 1 |
| Bed nucleus of the stria terminalis medial division anterior part | 29 | 2 | | Intermediate nucleus of the lateral lemniscus | 13 | 3 |
| Zona incerta caudal part | 28 | 5 | | Facial nucleus dorsal intermediate subnucleus | 13 | 0 |
| Septohippocampal nucleus | 28 | 3 | | Dorsal raphe nucleus interfascicular part | 13 | 1 |
| Primary visual cortex binocular area | 28 | 8 | | Deep white layer of the superior colliculus | 13 | 2 |
| Nucleus of the lateral olfactory tract layer 3 | 28 | 2 | | Amygdaloid intramedullary gray | 13 | 0 |
| Lateroanterior hypothalamic nucleus | 27 | 0 | | Supragenual nucleus | 12 | 0 |
| Inferior olive dorsal nucleus | 27 | 5 | | Retrosplenial granular a cortex layers | 12 | 3 |
| Facial nucleus lateral subnucleus | 27 | 4 | | Parvicellular reticular nucleus alpha part | 12 | 1 |
| Ventromedial preoptic nucleus | 26 | 2 | | Parabigeminal nucleus | 12 | 1 |
| Septohypothalamic nucleus | 26 | 6 | | Nucleus Y | 12 | 4 |
| Parastriatal nucleus | 26 | 3 | | Inferior olive cap of Kooy of the medial nucleus | 12 | 0 |
| Nucleus of the posterior commissure | 26 | 3 | | Arcuate nucleus lateral part | 12 | 1 |
| Interpeduncular nucleus caudal subnucleus | 26 | 5 | | Ambiguus nucleus loose part | 12 | 0 |
| Gracile nucleus principal part | 26 | 0 | | Retrosplenial granular b cortex layers | 11 | 3 |
| Boetzinger complex | 26 | 1 | | Nucleus ofoller | 11 | 0 |
| Medial geniculate nucleus dorsal part | 25 | 4 | | Medial tuberal nucleus | 11 | 0 |
| Medial division of the sublenticular extended amygdala | 25 | 2 | | Magnocellular nucleus of the posterior commissure | 11 | 1 |
| Interpeduncular nucleus lateral subnucleus | 25 | 7 | | Lacunosum molecular layer of the hippocampus | 11 | 0 |
| Zona incerta rostral part | 24 | 2 | | Facial nucleus dorsolateral subnucleus | 11 | 0 |
| Parapyramidal nucleus | 24 | 0 | | Dorsal tegmental nucleus central part | 11 | 1 |
| Nucleus of the trapezoid body | 24 | 6 | | Bed nucleus of the stria terminalis lateral division juxtacapsular part | 11 | 0 |
| Dorsomedial tegmental area | 24 | 0 | | Posterodorsal tegmental nucleus | 10 | 0 |
| Bed nucleus of the stria terminalis medial division posterior part | 24 | 4 | | Piriform cortex layer 3 | 10 | 0 |
| Ventro anterior thalamic nucleus | 23 | 5 | | Nucleus X | 10 | 0 |
| Ventral intermediate entorhinal cortex | 23 | 3 | | Laterodorsal thalamic nucleus ventrolateral part | 10 | 1 |
| Paratrigeminal nucleus | 23 | 0 | | A11 dopamine cells | 10 | 0 |
| Oval paracentral thalamic nucleus | 23 | 0 | | Supraoculomotor cap | 9 | 1 |
| Ventral nucleus of the lateral lemniscus | 22 | 7 | | Primary visual cortex monocular area | 9 | 3 |
| Superior salivatory nucleus | 22 | 0 | | Piriform cortex layer 2 | 9 | 1 |
| Superior paraolivary nucleus | 22 | 7 | | Nucleus of the lateral olfactory tract layer 1 | 9 | 0 |
| Rostral interstitial nucleus of medial longitudinal fasciculus | 22 | 2 | | Nucleus of the brachium of the inferior colliculus | 9 | 0 |
| Retrochiasmatic area lateral part | 22 | 1 | | Medial preoptic nucleus central part | 9 | 0 |
| Medial geniculate nucleus ventral part | 22 | 4 | | Magnocellular nucleus of the lateral hypothalamus | 9 | 1 |
| Lateral hypothalamic area [Region of tuber cinereum] | 22 | 4 | | Amygdalohippocampal area anterolateral part | 9 | 0 |
| Dorsomedial hypothalamic nucleus ventral part | 22 | 4 | | A13 dopamine cells | 9 | 0 |
| Ambiguus nucleus compact part | 22 | 1 | | Posteromedian thalamic nucleus | 8 | 1 |
| Supracapsular bed nucleus of the stria terminalis lateral part | 21 | 2 | | Nucleus of the solitary tract gelatinous part | 8 | 0 |
| Paraventricular hypothalamic nucleus posterior part | 21 | 0 | | Mitral cell layer of the accessory olfactory bulb | 8 | 1 |
| Lateral posterior thalamic nucleus mediorostral part | 21 | 4 | | Inferior salivatory nucleus | 8 | 0 |
| Dorsal nucleus of the lateral lemniscus | 21 | 6 | | Central gray alpha part | 8 | 1 |
| Caudal ventrolateral medulla lateral part | 21 | 0 | | Ventrolateral hypothalamic nucleus | 7 | 0 |
| Posterior pretectal nucleus | 20 | 1 | | Ventral reuniens thalamic nucleus | 7 | 1 |
| Optic nerve layer of the superior colliculus | 20 | 3 | | Suprachiasmatic nucleus dorsolateral part | 7 | 0 |
| Medial superior olive | 20 | 5 | | Subpostrema area | 7 | 0 |
| Medial pretectal nucleus | 20 | 3 | | Paraventricular hypothalamic nucleus ventral part | 7 | 0 |

| Region | Σ | Reci | Marker | Region | Σ | Reci |
|---|---|---|---|---|---|---|
| Lateral superior olive | 20 | 5 | | Intermediate interstitial nucleus of the medial longitudinal fasciculus | 7 | 1 |
| Superficial gray layer of the superior colliculus | 19 | 2 | | Intercalated amygdaloid nucleus main part | 7 | 0 |
| Subpeduncular tegmental nucleus | 19 | 1 | | Granule cell layer of the accessory olfactory bulb | 7 | 1 |
| Nucleus of the solitary tract ventral part | 19 | 1 | | Dorsal peduncular pontine nucleus | 7 | 0 |
| Nucleus of the solitary tract dorsolateral part | 19 | 0 | | Commissural nucleus of the inferior colliculus | 7 | 2 |
| Nucleus of the optic tract | 19 | 4 | | Amygdalohippocampal area posteromedial part | 7 | 0 |
| Interpeduncular nucleus apical subnucleus | 19 | 5 | | Xiphoid thalamic nucleus | 6 | 0 |
| Parasolitary nucleus | 18 | 1 | | Ventral geniculate nucleus | 6 | 0 |
| Paramedian reticular nucleus | 18 | 0 | | Substantia innominata basal part | 6 | 0 |
| Nucleus of the solitary tract interstitial part | 18 | 0 | | Nucleus of the stria medullaris | 6 | 0 |
| Medial preoptic nucleus medial part | 18 | 2 | | Nucleus circularis | 6 | 0 |
| Laterodorsal thalamic nucleus dorsomedial part | 6 | 0 | | Piriform cortex layer 1a | 1 | 0 |
| Lateral stripe of the striatum | 6 | 1 | | Pineal stalk | 1 | 0 |
| Lateral reticular nucleus subtrigeminal part | 6 | 0 | | Periolivary area | 1 | 0 |
| Lateral posterior thalamic nucleus laterocaudal part | 6 | 1 | | Parachochlear glial substance | 1 | 0 |
| Interpeduncular nucleus dorsomedial subnucleus | 6 | 0 | | P1 periaqueductal gray | 1 | 0 |
| Inferior olive subnucleus C of medial nucleus | 6 | 0 | | Olfactory ventricle | 1 | 0 |
| Ethmoid thalamic nucleus | 6 | 0 | | Oculomotor nucleus parvicellular part | 1 | 0 |
| Dorsal part of claustrum | 6 | 0 | | Nucleus of the spinal accessory nerve | 1 | 0 |
| Dorsal cochlear nucleus fusiform layer | 6 | 0 | | Nucleus of the central acoustic tract | 1 | 0 |
| Supraoptic nucleus retrochiasmatic part | 5 | 0 | | Navicular nucleus of the basal forebrain | 1 | 0 |
| Supraoculomotor periaqueductal gray | 5 | 0 | | Lateral paragigantocellular nucleus external part | 1 | 0 |
| Submedius thalamic nucleus ventral part | 5 | 0 | | Lamina terminalis | 1 | 0 |
| Submedius thalamic nucleus dorsal part | 5 | 0 | | Juxtaolivary nucleus | 1 | 0 |
| Secondary auditory cortex ventral area | 5 | 0 | | Islands of Calleja major island | 1 | 0 |
| Perifacial zone | 5 | 1 | | Interstitial nc. of the decussation of the superior cerebellar peduncle | 1 | 0 |
| Paraventricular hypothal. nc. posterior magnocellular part | 5 | 0 | | Internal medullary lamina | 1 | 0 |
| Paraventricular hypothalamic nucleus dorsal cap | 5 | 0 | | Fasciola cinereum | 1 | 0 |
| Nucleus of origin of efferents of the vestibular nerve | 5 | 0 | | Facial nucleus ventromedial subnucleus | 1 | 0 |
| Matrix region of the medulla | 5 | 0 | | Facial nucleus ventral intermediate subnucleus | 1 | 0 |
| Marginal zone of the medial geniculate | 5 | 0 | | Facial nucleus dorsomedial subnucleus | 1 | 0 |
| Lateral posterior thalamic nucleus mediocaudal part | 5 | 0 | | F cell group of the vestibular complex | 1 | 0 |
| Intercalated nucleus of the medulla | 5 | 0 | | External cortex of the inferior colliculus layer 3 | 1 | 0 |
| Dorsomedial hypothalamic nucleus compact part | 5 | 0 | | Episupraoptic nucleus | 1 | 0 |
| Cuneiforme nucleus dorsal part | 5 | 0 | | Epipeduncular nucleus | 1 | 0 |
| Bed nucleus of the anterior commissure | 5 | 0 | | Dorsal transition zone | 1 | 0 |
| Ventral part of claustrum | 4 | 1 | | Dorsal cochlear nucleus superficial layer | 1 | 0 |
| Terete hypothalamic nucleus | 4 | 0 | | Dorsal cochlear nucleus deep core | 1 | 0 |
| Subcommissural organ | 4 | 0 | | Cuneiforme nucleus ventral part | 1 | 0 |
| Subcoeruleus nucleus alpha part | 4 | 0 | | Cortex amygdala transition zone layer 1 | 1 | 0 |
| Striohypothalamic nucleus | 4 | 0 | | Caudal interstitial nucleus of the medial longitudinal fasciculus | 1 | 0 |
| Secondary visual cortex mediomedial area | 4 | 0 | | Bed nucleus of the stria terminalis anterior medial part medial subpart | 1 | 0 |
| Secondary visual cortex mediolateral area | 4 | 1 | | Bed nucleus of the stria terminalis anterior medial part lateral subpart | 1 | 0 |
| Sagulum nucleus | 4 | 1 | | Area periventricularis hypothalamica communis | 1 | 0 |
| Posterodorsal preoptic nucleus | 4 | 0 | | Arcuate hypothalamic nucleus medial posterior part | 1 | 0 |
| Paratrochlear nucleus | 4 | 0 | | Amygdalohippocampal area posterolateral part | 1 | 0 |
| Pararubral nucleus | 4 | 0 | | | | |

| Region | Σ | Reci | Marker | Region | Σ | Reci |
|---|---|---|---|---|---|---|
| Paraabducens nucleus | 4 | 1 | | Caudomedial entorhinal cortex | 2 | 0 |
| Olfactory tubercle plexiform layer | 4 | 0 | | Arcuate nucleus dorsal part | 2 | 0 |
| Medioventral periolivary nucleus | 4 | 1 | | Arcuate hypothalamic nucleus lateroposterior part | 2 | 0 |
| Lateral paragigantocellular nucleus alpha part | 4 | 0 | | Angular thalamic nucleus | 2 | 0 |
| Inferior olive subnucleus A of medial nucleus | 4 | 0 | | Zona limitans | 1 | 0 |
| External cortex of the inferior colliculus layer 2 | 4 | 0 | | Ventral cochlear nucleus anterior part | 1 | 0 |
| Dorsal tegmental nucleus pericentral part | 4 | 0 | | Trigeminal solitary transition zone | 1 | 0 |
| Anterior pretectal nucleus dorsal part | 4 | 0 | | Suprachiasmatic nucleus ventromedial part | 1 | 0 |
| Ventral cochlear nucleus posterior part | 3 | 0 | | Strial part of the preoptic area | 1 | 0 |
| Supracapsular bed nucleus of the stria terminalis medial part | 3 | 0 | | Rhabdoid nucleus | 1 | 0 |
| Retroparafascicular nucleus | 3 | 0 | | Retrosplenial granular cortex caudal part | 1 | 0 |
| Retroethmoid nucleus | 3 | 0 | | Retrolemniscal nucleus | 1 | 0 |
| Olfactory tubercle densocellular layer | 3 | 0 | | Reticulotegmental nucleus of the pons lateral part | 1 | 0 |
| Microcellular tegmental nucleus | 3 | 1 | | Reticular thalamic nucleus prosomere 1 | 1 | 0 |
| Medial eminence internal layer | 3 | 0 | | Precuneiform area | 1 | 0 |
| Lateroventral periolivary nucleus | 3 | 0 | | Posterodorsal raphe nuclei | 1 | 0 |
| Laterodorsal tegmental nucleus ventral part | 3 | 0 | | | | |
| Isthmic reticular formation | 3 | 0 | | | | |
| Interstitial nucleus of the vestibulocochlear nerve | 3 | 0 | | | | |
| Inferior olive subnucleus B of medial nucleus | 3 | 0 | | | | |
| Inferior olive dorsomedial cell group | 3 | 1 | | | | |
| Glomerular layer accessory olfactory bulb | 3 | 0 | | | | |
| Gemini hypothalamic nucleus | 3 | 0 | | | | |
| Bed nucleus of the stria terminalis lateral division intermediate part | 3 | 0 | | | | |
| Anterior tegmental nucleus | 3 | 0 | | | | |
| Anterior pretectal nucleus ventral part | 3 | 0 | | | | |
| Vestibulocerebellar nucleus | 2 | 0 | | | | |
| Ventral cochlear nucleus granule cell layer | 2 | 0 | | | | |
| Superior colliculus zonal layer | 2 | 0 | | | | |
| Substantia gelatinosa of the trigeminal sensory nuclear complex | 2 | 0 | | | | |
| Submedius thalamic nucleus parvicellular part | 2 | 0 | | | | |
| Subbrachial nucleus | 2 | 0 | | | | |
| Sphenoid nucleus | 2 | 0 | | | | |
| Sensory root of the trigeminal nerve | 2 | 0 | | | | |
| Retroreuniens area | 2 | 0 | | | | |
| Reticulotegmental nucleus of the pons pericentral part | 2 | 0 | | | | |
| Piriform cortex layer 1b | 2 | 1 | | | | |
| Nucleus Z | 2 | 0 | | | | |
| Nucleus O | 2 | 0 | | | | |
| Medial entorhinal cortex rostral part | 2 | 0 | | | | |
| Linear nucleus of the medulla | 2 | 0 | | | | |
| Lambdoid septal zone | 2 | 0 | | | | |
| Intramedullary thalamic area | 2 | 0 | | | | |
| Interpeduncular nucleus dorsolateral subnucleus | 2 | 0 | | | | |
| External plexiform layer of the accessory olfactory bulb | 2 | 0 | | | | |
| External cortex of the inferior colliculus layer 1 | 2 | 0 | | | | |
| Dorsal periolivary region | 2 | 0 | | | | |
