# Supplementary material for: Connectome-based prediction of functional impairment in experimental stroke models
Source: PLoS One. 2024 Dec 19;19(12):e0310743. doi: 10.1371/journal.pone.0310743 (PMC11658581; doi:10.1371/journal.pone.0310743)
Supplement: S5 Table — Connections of ICH lesioned regions, functionally defined regions and control regions without functional definitions. (PDF) [file pone.0310743.s011.pdf]

S4 Table. Overview of all connections of ICH lesioned regions. Connections of ICH lesioned regions, functionally defined regions and control regions without functional definitions.

Region	Σ	Reci	Marker	Region	Σ	Reci
Perirhinal cortex	10	3	Learn	Gigantocellular reticular nucleus alpha part	2	0
Subiculum	7	1	Learn	Parvicellular reticular nucleus alpha part	2	0
Field CA1 of hippocampus	6	1	Learn	C2 adrenaline cells	2	0
Lateral entorhinal cortex	4	3	Learn	C3 adrenaline cells	2	1
Dentate gyrus	3	0	Learn	Area postrema	2	2
Parasubiculum	3	0	Learn	Spinal trigeminal nucleus	2	0
Cingulate cortex area 1	3	1	Learn	Nucleus of the solitary tract commissural part	2	2
Cingulate cortex area 2	3	0	Learn	Nucleus of the solitary tract dorsolateral part	2	0
Interoanteromedial thalamic nucleus	2	0	Learn	Nucleus of the solitary tract dorsomedial part	2	0
Rhomboid nucleus	2	0	Learn	Nucleus of the solitary tract intermediate part	2	0
Subparafascicular thalamic nucleus rostral part	2	0	Learn	Nucleus of the solitary tract medial part	2	2
Mammillary body	1	0	Learn	Nucleus of the solitary tract ventral part	2	0
Field CA3 of hippocampus	1	0	Learn	Dorsal raphe nucleus dorsal part	2	0
Postrhinal cortex	1	0	Learn	Dorsal raphe nucleus ventral part	2	0
Medial agranular prefrontal cortex	15	6	Mot	Pontine raphe nucleus	2	0
Substantia nigra compact part	11	3	Mot	Medial septal nucleus	2	0
Substantia nigra reticular part	11	2	Mot	Substantia innominata basal part	2	0
Lateral agranular prefrontal cortex	10	7	Mot	Magnocellular preoptic nucleus	2	0
Caudate putamen	9	3	Mot	Dorsal tenia tecta	2	0
Ventrolateral thalamic nucleus	6	0	Mot	Lateroanterior hypothalamic nucleus	2	0
Medial globus pallidus	5	0	Mot	Ventromedial hypothalamic nucleus dorsomedial part	2	0
Lateral globus pallidus	4	1	Mot	Paraventricular hypothalamic nucleus lateral magnocellular part	2	0
Subthalamic nucleus	4	1	Mot	Supraoptic nucleus retrochiasmatic part	2	0
Lateral hypothalamic area	16	7		Anterior hypothalamic area central part	2	0
Prelimbic cortex	15	5		Posterior limitans thalamic nucleus	2	0
Infralimbic cortex	14	5		Posterior thalamic nuclear group triangular part	2	0
Agranular insular cortex dorsal part	14	1		Intermediodorsal thalamic nucleus	2	2
Locus coeruleus	12	3		Mediodorsal thalamic nucleus medial part	2	1
Ventral pallidum	12	1		Zona incerta dorsal part	2	1
Dysgranular insular cortex	12	0		Ventrolateral part of the lateral nucleus	2	0
Pedunculopontine tegmental nucleus	11	4		Ventromedial part of the lateral nucleus	2	0
Primary somatosensory cortex	11	6		Bed nucleus of the stria terminalis lateral division juxtacapsular part	2	0
Secondary somatosensory cortex	11	3		Bed nucleus of the stria terminalis medial division posterior part	2	0
Substantia nigra lateral part	10	4		Bed nucleus of the stria terminalis medial division ventral part	2	0
Parabrachial nucleus medial	10	2		Medial amygdaloid nucleus posterodorsal part	2	0
A8 dopamine cells retrorubral group	10	1		Ventral basolateral nucleus	2	0
Posterior basolateral nucleus	10	5		Dorsolateral entorhinal cortex	2	0
Agranular insular cortex posterior part	10	2		Dorsolateral orbital cortex	2	0
Granular insular cortex	10	1		Medial geniculate nucleus dorsal part	2	0
Lateral orbital cortex	10	0		Medial geniculate nucleus medial part	2	1
Central medial thalamic nucleus	9	4		Medial geniculate nucleus ventral part	2	0
Paraventricular thalamic nucleus	9	1		Central gray alpha part	1	0
Anterior basomedial nucleus	9	7		Parabigeminal nucleus	1	0
Amygdalopiriform transition area	9	5		Dorsomedial periaqueductal gray	1	0
Medial orbital cortex	9	2		Red nucleus magnocellular part	1	0

Region	Σ	Reci	Marker	Region	Σ	Reci
Caudal linear nucleus of the raphe	8	0		Red nucleus parvicellular part	1	0
Lateral preoptic area	8	0		Interfascicular nucleus	1	0
Parafascicular thalamic nucleus	8	4		Paranigral nucleus	1	0
Paratenial thalamic nucleus	8	0		Dorsomedial tegmental area	1	0
Agranular insular cortex ventral part	8	2		Perifacial zone	1	0
Ventrolateral periaqueductal gray	7	2		Peritrigeminal zone	1	0
Retrorubral nucleus	7	1		Nucleus of the solitary tract central part	1	0
Pontine reticular nucleus caudal part	7	0		Nucleus of the solitary tract gelatinous part	1	0
Lateral parabrachial nucleus	7	3		Nucleus of the solitary tract rostrolateral part	1	0
Median raphe nucleus	7	2		Dorsal raphe nucleus lateral wing	1	0
Cuneate nucleus	7	0		Paramedian raphe nucleus	1	0
Posterior basomedial nucleus	7	1		Raphe interpositus nucleus	1	0
Anterior amygdaloid area	7	3		Raphe pallidus nucleus	1	0
Anterior cortical amygdaloid nucleus	7	5		Caudal ventrolateral medulla lateral part	1	0
Medial amygdaloid nucleus anterodorsal part	7	0		A1 noradrenergic cells	1	0
Frontal cortex area 3	7	0		Caudoventral reticular nucleus	1	0
Primary visual cortex	7	2		Gracile nucleus principal part	1	0
Ventral orbital cortex	7	2		Superior salivatory nucleus	1	0
Dorsolateral periaqueductal gray	6	0		Nucleus of the vertical limb of the diagonal band	1	0
Peripeduncular nucleus	6	1		Median preoptic nucleus	1	0
Superior vestibular nucleus	6	1		Medial preoptic area	1	0
Paraventricular hypothalamic nucleus anterior parvicellular part	6	1		Ventral tenia tecta	1	1
Dorsolateral part of the lateral nucleus	6	1		Paraventricular hypothalamic nucleus ventral part	1	0
Bed nucleus of the stria terminalis lateral division dorsal part	6	2		Retrochiasmatic area lateral part	1	0
Bed nucleus of the stria terminalis lateral division ventral part	6	1		Retroethmoid nucleus	1	0
Posterior amygdaloid nucleus	6	3		Retroparafascicular nucleus	1	0
Posterolateral cortical nucleus	6	2		Mediodorsal thalamic nucleus central part	1	0
Medial amygdaloid nucleus posteroventral part	6	0		Subfornical organ	1	0
Parietal association cortex	6	3		Ventral posterior thalamic nucleus parvicellular part	1	0
Flocculus	5	0		Ethmoid thalamic nucleus	1	0
Pontine reticular nucleus oral part	5	0		Laterodorsal thalamic nucleus dorsomedial part	1	0
Koelliker Fuse nucleus	5	1		Nucleus of the stria medullaris	1	0
Principal sensory trigeminal nucleus	5	1		Posteromedian thalamic nucleus	1	0
Lateral vestibular nucleus	5	0		Nucleus of the fields of Forel	1	0
Dorsal motor nucleus of vagus	5	1		Zona incerta caudal part	1	0
Rostral linear nucleus of the raphe	5	0		Zona incerta ventral part	1	0
Rhomboid thalamic nucleus	5	0		Lateral habenular nucleus lateral part	1	0
Reuniens thalamic nucleus	5	1		Medial habenular nucleus	1	0
Paraventricular thalamic nucleus anterior part	5	0		Bed nucleus of the stria terminalis medial division posterointermediate part	1	0
Paraventricular thalamic nucleus posterior part	5	0		Bed nucleus of the stria terminalis dorsal nucleus	1	0
Posterior intralaminar thalamic nucleus	5	2		Nucleus of the lateral olfactory tract layer 1	1	0
Bed nucleus of the stria terminalis fusiform part	5	3		Nucleus of the lateral olfactory tract layer 3	1	0
Supracapsular bed nucleus of the stria terminalis lateral part	5	1		Amygdalohippocampal area anterolateral part	1	0
Central division of sublenticular extended amygdala	5	0		Amygdalohippocampal area posteromedial part	1	0
Posteromedial cortical nucleus	5	2		Lateral accumbens shell	1	0
Intercalated nuclei of the amygdala	5	0		Dorsal part of claustrum	1	0
Ectorhinal cortex	5	2		Septohippocampal nucleus	1	0
Dorsal peduncular cortex	5	0		Postsubiculum	1	0
Secondary visual cortex lateral area	5	0		Dorsal intermediate entorhinal cortex	1	0

Region	Σ	Reci	Marker	Region	Σ	Reci
Lateral periaqueductal gray	4	0		Piriform cortex layer 3	1	0
Parabrachial pigmented nucleus	4	0		Retrosplenial dorsal	1	0
Edinger Westphal nucleus	4	0		Temporal association cortex 1	1	0
Spinal vestibular nucleus	4	0		Primary auditory cortex	1	0
Medial vestibular nucleus	4	1		Precommissural nucleus	1	0
Raphe magnus nucleus	4	0		Ventral lateral geniculate nucleus	1	0
Ventromedial hypothalamic nucleus central part	4	0		Interpeduncular nucleus apical subnucleus	1	0
Suprageniculate thalamic nucleus	4	1		Interpeduncular nucleus caudal subnucleus	1	1
Bed nucleus of the stria terminalis intraamygdaloid division	4	0		Interpeduncular nucleus intermediate subnucleus	1	0
Bed nucleus of the stria terminalis lateral division posterior part	4	0		Interpeduncular nucleus rostral subnucleus	1	0
Bed nucleus of the stria terminalis medial division anterior part	4	0		Lateral septal nucleus ventral part	1	0
Central gray pons part	3	0				
Nucleus of Darkschewitsch	3	0				
Subpeduncular tegmental nucleus	3	0				
Interstitial nucleus of Cajal	3	0				
A5 noradrenaline cells	3	0				
Prepositus nucleus	3	0				
Subcoeruleus nucleus dorsal part	3	0				
Subcoeruleus nucleus ventral part	3	0				
Paratrigeminal nucleus	3	0				
Supratrigeminal nucleus	3	0				
Mesencephalic trigeminal nucleus	3	2				
Nucleus of the solitary tract ventrolateral part	3	0				
Dorsal raphe nucleus caudal part	3	0				
A2 noradrenergic cells	3	0				
Dorsal paragigantocellular nucleus	3	0				
Nucleus of the horizontal limb of the diagonal band	3	0				
Dorsal hypothalamic area	3	0				
Parastriatal nucleus	3	0				
Posterior hypothalamic nucleus	3	0				
Ventromedial hypothalamic nucleus ventrolateral part	3	0				
Nucleus of the lateral olfactory tract layer 2	3	0				
Medial amygdaloid nucleus anteroventral part	3	0				
Bed nucleus of the accessory olfactory tract	3	0				
Accumbens nucleus core	3	0				
Ventral intermediate entorhinal cortex	3	0				
Anterior olfactory nucleus	3	1				
Ventral tegmental area rostral part	2	0				
Ventral tegmental nucleus	2	0				
A7 noradrenaline cells	2	0				
Barringtons nucleus	2	0				
