# Supplementary material for: Connectome-based prediction of functional impairment in experimental stroke models
Source: PLoS One. 2024 Dec 19;19(12):e0310743. doi: 10.1371/journal.pone.0310743 (PMC11658581; doi:10.1371/journal.pone.0310743)
Supplement: S6 Table — This table (subset stats table) allows comparison of several parameters and matrices like the connectivity matching matrix (CMIAll), GTOM and FHN of the lesioned regions (2nd, 8th, 16th … column) of the ICH connectome with non lesioned regions (first colum, all rows). The lower table displays a clipping of the lesioned regions basal nucleus Meynert (B_R) and anteroventral thalamic nucleus (AV_R). The non lesioned regions were sorted by the CMIAll parameter: The lateral enthorinal cortex has the largest CMIAll value (from all non lesioned regions) with the basal nucleus Meynert. (PDF) [file pone.0310743.s012.pdf]

S5 Table. Identification of ICH lesioned regions strongly connected with functionally defined regions. This table (subset stats table) allows comparison of several parameters and matrices like the connectivity matching matrix (CMI_{All}), GTOM and FHN of the lesioned regions (2nd, 8th, 16th ... column) of the ICH connectome with non lesioned regions (first colum, all rows). The lower table displays a clipping of the lesioned regions basal nucleus Meynert (B_R) and anteroventral thalamic nucleus (AV_R). The non lesioned regions were sorted by the CMI_{All} parameter: The lateral enthorinal cortex has the largest CMI_{All} value (from all non lesioned regions) with the basal nucleus Meynert.

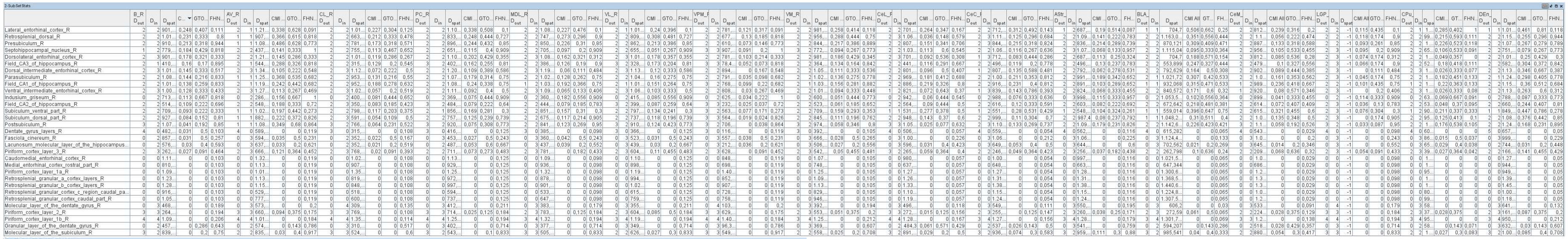

2-SubSetStats

	B_R D_{out}	D_{in}	D_{spat}	C...	GTO...	FHN...	AV_R D_{out}	D_{in}	D_{spat}	CMI ...	GTO...	FHN...
Lateral_entorhinal_cortex_R	2	2	901,...	0,248	0,407	0,111	2	1	1.21...	0,338	0,628	0,091
Retrosplenial_dorsal_R	2	2	1.01...	0,231	0,333	0,8	1	1	907,...	0,366	0,615	0,818
Presubiculum_R	2	2	910,...	0,213	0,318	0,944	1	1	1.08...	0,486	0,628	0,773
Septohippocampal_nucleus_R	1	2	779,...	0,184	0,429	0,818	2	2	437,...	0,141	0,333	1
Dorsolateral_entorhinal_cortex_R	2	3	901,...	0,178	0,321	0,333	2	2	1.21...	0,145	0,286	0,333
Field_CA3_of_hippocampus_R	2	1	410,...	0,16	0,17	0,895	2	1	544,...	0,288	0,326	0,818
Dorsal_intermediate_entorhinal_cortex_R	2	3	1.01...	0,145	0,333	0,517	2	3	1.34...	0,105	0,222	0,548
Parasubiculum_R	2	2	1.08...	0,144	0,216	0,833	1	1	1.25...	0,368	0,535	0,682
Field_CA1_of_hippocampus_R	2	2	1.01...	0,138	0,259	0,833	2	1	1.19...	0,242	0,442	0,682
Ventral_intermediate_entorhinal_cortex_R	2	3	1.00...	0,128	0,333	0,433	2	3	1.27...	0,113	0,267	0,469
Indusium_griseum_R	0	2	713,...	0,113	0,667	0,818	0	2	286,...	0,156	0,667	1
Field_CA2_of_hippocampus_R	2	2	514,...	0,109	0,222	0,696	2	2	548,...	0,188	0,333	0,72
Subiculum_ventral_part_R	2	2	709,...	0,093	0,222	0,333	1	1	1.02...	0,197	0,442	0,273
Subiculum_dorsal_part_R	2	2	927,...	0,084	0,152	0,81	1	1	882,...	0,222	0,372	0,826
Postsubiculum_R	3	2	1.07...	0,041	0,192	0,85	1	1	1.08...	0,349	0,68	0,864
Dentate_gyrus_layers_R	4	0	482,...	0,031	0,5	0,103	4	0	589,...	0	0	0,119
Fasciola_cinereum_R	0	2	857,...	0,031	0,5	0,257	0	3	594,...	0,035	0,5	0,231
Lacunosum_molecular_layer_of_the_hippocampus...	0	2	576,...	0,03	0,4	0,593	0	3	637,...	0,033	0,2	0,621
Piriform_cortex_layer_3_R	2	3	262,...	0,027	0,091	0,464	2	3	666,...	0,121	0,364	0,452
Caudomedial_entorhinal_cortex_R	0	0	1.11...	0	0	0,103	0	0	1.32...	0	0	0,119
Medial_entorhinal_cortex_rostral_part_R	0	0	810,...	0	0	0,103	0	0	1.13...	0	0	0,119
Piriform_cortex_layer_1a_R	0	0	1.09...	0	0	0,103	0	0	1.01...	0	0	0,119
Retrosplenial_granular_a_cortex_layers_R	0	0	1.23...	0	0	0,103	0	0	1.13...	0	0	0,119
Retrosplenial_granular_b_cortex_layers_R	0	0	1.28...	0	0	0,103	0	0	1.15...	0	0	0,119
Retrosplenial_granular_cortex_c_region_caudal_pa...	0	0	916,...	0	0	0,103	0	0	529,...	0	0	0,119
Retrosplenial_granular_cortex_caudal_part_R	0	0	1.05...	0	0	0,103	0	0	777,...	0	0	0,119
Molecular_layer_of_the_dentate_gyrus_R	0	3	468,...	0	0	0,189	0	3	573,...	0	0	0,2
Piriform_cortex_layer_2_R	3	3	264,...	0	0	0,194	3	3	660,...	0,094	0,375	0,175
Piriform_cortex_layer_1b_R	4	4	1.09...	0	0	0,206	4	4	1.01...	0	0	0,184
Granular_layer_of_the_dentate_gyrus_R	0	2	457,...	0	0,286	0,643	0	2	574,...	0	0,143	0,786
Molecular_layer_of_the_subiculum_R	3	2	839,...	0	0,2	0,75	2	2	835,...	0,03	0,4	0,917
