# Supplementary material for: Connectome-based prediction of functional impairment in experimental stroke models
Source: PLoS One. 2024 Dec 19;19(12):e0310743. doi: 10.1371/journal.pone.0310743 (PMC11658581; doi:10.1371/journal.pone.0310743)
Supplement: S7 Table — Connections of sMCAO lesioned regions, functionally defined regions and control regions without functional definitions. (PDF) [file pone.0310743.s013.pdf]

S6 Table. Overview of all connections of sMCAO lesioned regions. Connections of sMCAO lesioned regions, functionally defined regions and control regions without functional definitions.

Region	Σ	Reci	Marker	Region	Σ	Reci
Perirhinal cortex	69	211	Learn	Ventromedial hypothalamic nucleus central part	20	85
Lateral entorhinal cortex	63	177	Learn	A8 dopamine cells retrorubral group	20	111
Field CA1 of hippocampus	49	184	Learn	Ventral posteromedial thalamic nucleus	20	77
Cingulate cortex area 1	33	105	Learn	Raphe pallidus nucleus	19	110
Rhomboid thalamic nucleus	28	146	Learn	Rostral ventral respiratory group	19	85
Postrhinal cortex	25	68	Learn	Posterior intralaminar thalamic nucleus	19	86
Presubiculum	24	87	Learn	Medial amygdaloid nucleus anterodorsal part	19	86
Cingulate cortex area 2	22	82	Learn	Retrorubral nucleus	18	79
Parasubiculum	21	87	Learn	Nucleus of the solitary tract medial part	18	83
Field CA3 of hippocampus	16	92	Learn	Dorsal hypothalamic area	18	106
Field CA2 of hippocampus	12	62	Learn	Ventromedial hypothalamic nucleus ventrolateral part	18	106
Interoanteromedial thalamic nucleus	7	53	Learn	Subiculum dorsal part	18	78
Caudate putamen	38	177	Mot	Temporal association cortex 1	18	79
Substantia nigra reticular part	22	112	Mot	Mediodorsal thalamic nucleus medial part	17	76
Ventrolateral thalamic nucleus	18	78	Mot	Subfornical organ	17	59
Medial globus pallidus	17	70	Mot	Retrosplenial dorsal	17	50
Pontine nuclei	11	83	Mot	Ventral lateral geniculate nucleus	17	71
Lateral hypothalamic area	154	459		Dorsal raphe nucleus lateral wing	16	86
Bed nucleus of the stria terminalis	112	323		Premammillary nucleus dorsal part	16	80
Central amygdaloid nucleus	111	320		Centrolateral thalamic nucleus	16	102
Motor regions	98	328		Medial amygdaloid nucleus posterodorsal part	16	56
Infralimbic cortex	96	280		Dorsolateral periaqueductal gray	15	124
Zona incerta	90	287		A5 noradrenaline cells	15	81
Locus coeruleus	82	307		Lateral vestibular nucleus	15	88
Prelimbic cortex	81	251		Nucleus of the solitary tract commissural part	14	74
Median raphe nucleus	80	281		Basal nucleus Meynert	14	89
Substantia innominata	75	234		Paraventricular hypothalamic nucleus anterior parvicellular part	14	84
Medial preoptic area and medial preoptic nucleus lateral and medial	72	244		Anterodorsal thalamic nucleus	14	59
Basolateral amygdaloid nucleus	72	231		Intermediodorsal thalamic nucleus	14	82
Agranular insular cortex	71	213		Paraventricular thalamic nucleus posterior part	14	90
Cortical amygdaloid nucleus	67	193		Medial amygdaloid nucleus posteroventral part	14	63
Reuniens thalamic nucleus	58	225		Central nucleus of the inferior colliculus	13	44
Paraventricular thalamic nucleus	57	196		Barringtons nucleus	13	74
Lateral parabrachial nucleus	56	201		Ventromedial hypothalamic nucleus dorsomedial part	13	73
Ventrolateral periaqueductal gray	53	202		Medial habenular nucleus	13	66
Pedunculopontine tegmental nucleus	51	190		Postsubiculum	13	46
Piriform cortex	50	172		Dorsal peduncular cortex	13	85
Anterior hypothalamic area	49	186		Precommissural nucleus	13	59
Claustrum	49	158		Medial geniculate nucleus medial part	13	65
Ventral pallidum	48	154		Substantia nigra lateral part	12	59
Primary somatosensory cortex	48	170		Spinal vestibular nucleus	12	78
Nucleus of the horizontal limb of the diagonal band	47	168		Dorsal motor nucleus of vagus	12	104
Lateral preoptic area	46	182		Ventral tenia tecta	12	38
Medial preoptic nucleus	46	160		Anterior hypothalamic area posterior part	12	51
Posterior hypothalamic nucleus	46	184		Mediodorsal thalamic nucleus lateral part	12	59
Reticular thalamic nucleus	46	170		Ventral posterolateral thalamic nucleus	12	69

Region	Σ	Reci	Marker	Region	Σ	Reci
Medial septal nucleus	45	170		Dorsolateral entorhinal cortex	12	49
Raphe magnus nucleus	44	186		Lateral septal nucleus intermediate part	12	70
Parafascicular thalamic nucleus	44	176		Gigantocellular reticular nucleus alpha part	11	65
Parietal association cortex	44	156		Area postrema	11	61
Nucleus of the vertical limb of the diagonal band	42	142		Ventrolateral preoptic nucleus	11	59
Medial orbital cortex	42	137		Lateral mammillary nucleus	11	68
Subiculum ventral part	40	147		Paracentral thalamic nucleus	11	97
Ectorhinal cortex	40	121		Nucleus of the solitary tract ventrolateral part	10	46
Edinger Westphal nucleus	39	187		Dorsal tenia tecta	10	43
Parabrachial nucleus medial	38	158		Vascular organ of the lamina terminalis	10	45
Spinal trigeminal nucleus	38	179		Paraventricular hypothalamic nucleus medial parvicellular part	10	65
Ventral orbital cortex	38	125		Suprageniculate thalamic nucleus	10	60
Subparafascicular thalamic nucleus	37	134		Mediodorsal thalamic nucleus central part	10	59
Juxtaparaventricular part [Lateral hypothalamic area]	36	121		Medial amygdaloid nucleus anteroventral part	10	44
Posterior basomedial nucleus	35	128		Triangular septal nucleus	10	45
Anterior amygdaloid area	35	133		Interfascicular nucleus	9	61
Amygdalopiriform transition area	34	112		Caudal linear nucleus of the raphe	9	65
Lateral orbital cortex	34	131		Peripeduncular nucleus	9	68
Anterior basomedial nucleus	33	125		Flocculus	9	72
Central medial thalamic nucleus	32	155		Bed nucleus of the accessory olfactory tract	9	33
Paratenial thalamic nucleus	32	126		Parasubthalamic nucleus	9	65
Medial vestibular nucleus	31	140		Primary visual cortex binocular area	9	31
Nucleus of the lateral olfactory tract	31	91		Dorsolateral orbital cortex	9	36
Primary visual cortex	31	109		Supratrigeminal nucleus	8	64
Central amygdaloid nucleus medial division	29	152		Median eminence	8	54
Granular insular cortex	29	119		Ventral endopiriform nucleus	8	40
Olfactory tubercle	29	133		Subparaventricular zone of the hypothalamus	8	56
Pontine reticular nucleus oral part	26	128		Supramammillar nucleus lateral part	8	74
Posteromedial cortical nucleus	26	84		Medial terminal nucleus of the accessory optic tract	8	43
Dysgranular insular cortex	26	115		Amygdalostriatal transition area	8	66
Magnocellular preoptic nucleus	25	103		Septofimbrial nucleus	8	50
Anteroventral thalamic nucleus	25	85		Ventral nucleus of the lateral lemniscus	7	22
Anterior olfactory nucleus	25	93		Rostral linear nucleus of the raphe	7	85
Prepositus nucleus	24	99		Pontine raphe nucleus	7	75
Pontine reticular nucleus caudal part	24	113		A1 noradrenergic cells	7	59
Median preoptic nucleus	24	103		Superior paraolivary nucleus	7	23
Secondary somatosensory cortex	24	96		Paraventricular thalamic nucleus anterior part	7	76
Lateral periaqueductal gray	23	117		Tuber cinereum area	7	51
Principal sensory trigeminal nucleus	23	121		Ventrolateral part of the lateral nucleus	7	50
Interstitial nucleus of the posterior limb of the anterior commissure	23	108		Ventromedial part of the lateral nucleus	7	55
Accumbens nucleus core	23	93		Intercalated nuclei of the amygdala	7	38
Lateral septal nucleus ventral part	23	86		Dorsal geniculate nucleus	7	37
Ventral tegmental nucleus	22	96		Interpeduncular nucleus lateral subnucleus	7	23
Superior vestibular nucleus	22	90		Interpeduncular nucleus rostral subnucleus	7	47
Premammillary nucleus ventral part	22	94		Olfactory cortex layers	7	30
Anteromedial thalamic nucleus	22	82		Dorsal nucleus of the lateral lemniscus	6	22

Region	Σ	Reci	Marker	Region	Σ	Reci
Ventromedial thalamic nucleus	22	118		Nucleus of Darkschewitsch	6	80
Secondary visual cortex lateral area	22	93		Dorsomedial periaqueductal gray	6	45
Raphe obscurus nucleus	21	92		Deep gray layer of the superior colliculus	6	30
Primary auditory cortex	21	73		Cerebellar lobule 10	6	61
Koelliker Fuse nucleus	20	111		Nucleus of the trapezoid body	6	25
Mesencephalic trigeminal nucleus	20	136		Nucleus of the solitary tract intermediate part	6	45
Dorsal raphe nucleus caudal part	20	110		Nucleus of the solitary tract lateral part	6	38
Dorsal raphe nucleus ventral part	6	53		Central gray alpha part	1	11
Paramedian raphe nucleus	6	34		Sagulum nucleus	1	4
Lateral hypothalamic area [Region of tuber cinereum]	6	26		Parabigeminal nucleus	1	13
Lateral habenular nucleus medial part	6	47		Red nucleus parvicellular part	1	13
Central division of sublenticular extended amygdala	6	34		Intermediate white layer of the superior colliculus	1	13
Dorsal intermediate entorhinal cortex	6	31		Microcellular tegmental nucleus	1	4
Lateral septal nucleus dorsal part	6	53		Subpeduncular tegmental nucleus	1	20
Central gray pons part	5	47		Dorsal tegmental nucleus central part	1	11
Dorsal cortex of the inferior colliculus	5	34		Cerebellar lobule 1	1	24
Paranigral nucleus	5	38		Cerebellar lobule 8	1	33
Rostroventrolateral reticular nucleus	5	43		Interposed cerebellar nucleus dorsolateral hump	1	4
Motor trigeminal nucleus	5	60		A7 noradrenaline cells	1	33
Nucleus Y	5	19		Intermediate interstitial nucleus of the medial longitudinal fasciculus	1	7
Lateral superior olive	5	20		Parvicellular reticular nucleus alpha part	1	11
Medial superior olive	5	20		C2 adrenaline cells	1	22
Dorsal paragigantocellular nucleus	5	60		Ambiguus nucleus compact part	1	22
Dorsomedial hypothalamic nucleus ventral part	5	25		Boetzinger complex	1	26
Supramammillar nucleus medial part	5	55		Paraabducens nucleus	1	4
Dorsolateral part of the lateral nucleus	5	58		Perifacial zone	1	5
Frontal cortex area 3	5	32		Peritrigeminal zone	1	14
Intergeniculate leaf	5	46		Nucleus of the solitary tract central part	1	19
Medial geniculate nucleus ventral part	5	24		Nucleus of the solitary tract dorsolateral part	1	22
Interpeduncular nucleus caudal subnucleus	5	26		Parasolitary nucleus	1	19
Septohypothalamic nucleus	5	29		Dorsal raphe nucleus interfascicular part	1	15
Medial paralemniscal nucleus	4	41		B9 serotonin cells	1	20
Interstitial nucleus of Cajal	4	53		Gracile nucleus principal part	1	27
Paraflocculus	4	63		Intercalated nucleus of the medulla	1	7
Cerebellar lobule 9ab	4	22		Medioventral periolivary nucleus	1	4
Cerebellar lobule 9c	4	21		Dorsal tuberomammillary nucleus	1	18
Lateral cerebellar nucleus parvicellular part	4	31		Magnocellular nucleus of the lateral hypothalamus	1	10
Gigantocellular reticular nucleus ventral part	4	37		Posterior hypothalamic area dorsal part	1	16
Medullary reticular nucleus ventrolateral part	4	54		Arcuate nucleus lateral part	1	14
Facial nucleus lateral subnucleus	4	27		Medial mammillary nucleus medial part	1	26
Nucleus of the solitary tract rostrolateral part	4	17		Medial mammillary nucleus median part	1	17
A2 noradrenergic cells	4	39		Supraoculomotor cap	1	9
Inferior olive dorsal nucleus	4	45		Posterior limitans thalamic nucleus	1	16
Olivary pretectal nucleus	4	31		Posterior thalamic nuclear group triangular part	1	17
Ventral tuberomammillary nucleus	4	49		Lateral posterior thalamic nucleus laterocaudal part	1	6
Medial mammillary nucleus lateral part	4	27		Laterodorsal thalamic nucleus ventrolateral part	1	9
Lateral posterior thalamic nucleus mediorostral part	4	21		Nucleus of the stria medullaris	1	7
Nucleus of the optic tract	4	20		Ventral reuniens thalamic nucleus	1	8
Intercalated masses	4	31		Lateral stripe of the striatum	1	9

Region	Σ	Reci	Marker	Region	Σ	Reci
Molecular layer of the subiculum	4	20		Dorsomedial hypothalamic nucleus dorsal part	2	151
Ventral intermediate entorhinal cortex	4	24		Posteromedian thalamic nucleus	2	11
Retrosplenial granular b cortex layers	4	14		Posterior pretectal nucleus	2	18
Nucleus of the posterior commissure	4	24		Lateral terminal nucleus of the accessory optic tract	2	16
edial geniculate nucleus dorsal part	4	27		Lateral accumbens shell	2	46
Interpeduncular nucleus apical subnucleus	4	16		Septohippocampal nucleus	2	32
Intermediate nucleus of the lateral lemniscus	3	14		Retrosplenial granular a cortex layers	2	13
Optic nerve layer of the superior colliculus	3	18		Mitral cell layer of the accessory olfactory bulb	2	11
Intermediate gray layer of the superior colliculus	3	32		Retrosplenial granular cortex c region caudal part	2	12
Substantia nigra compact part dorsal tier	3	24		Secondary visual cortex mediolateral area	1	4
Substantia nigra compact part medial tier	3	23		Magnocellular nucleus of the posterior commissure	1	11
Medial cerebellar nucleus dorsolateral protuberance	3	31		Granule cell layer of the accessory olfactory bulb	1	11
C1 adrenaline cells	3	40		Dorsal part of claustrum	1	8
Nucleus of the solitary tract ventral part	3	22				
Dorsal raphe nucleus dorsal part	3	60				
Cuneate nucleus	3	67				
Parastriatal nucleus	3	26				
Retrochiasmatic area lateral part	3	26				
Ventro anterior thalamic nucleus	3	19				
Ventral posterior thalamic nucleus parvicellular part	3	31				
Lateral posterior thalamic nucleus laterorostral part	3	17				
Lateral habenular nucleus lateral part	3	35				
Medial pretectal nucleus	3	21				
Medial division of the sublenticular extended amygdala	3	28				
Secondary auditory cortex	3	25				
Primary visual cortex monocular area	3	12				
Interpeduncular nucleus intermediate subnucleus	3	18				
Medial accessory oculomotor nucleus	2	12				
Rostral interstitial nucleus of medial longitudinal fasciculus	2	21				
Prerubral field	2	14				
Red nucleus magnocellular part	2	15				
Commissural nucleus of the inferior colliculus	2	7				
Superficial gray layer of the superior colliculus	2	17				
Deep white layer of the superior colliculus	2	13				
Substantia nigra compact part ventral tier	2	29				
Ventral tegmental area rostral part	2	45				
Parabrachial pigmented nucleus	2	43				
Cerebellar lobule 7	2	34				
Cerebellar lobule 6a	2	28				
Cerebellar lobule 6b	2	26				
Cerebellar lobule 6c	2	26				
Subcoeruleus nucleus dorsal part	2	45				
C3 adrenaline cells	2	21				
Principal hypoglossal nucleus	2	35				
Nucleus of the solitary tract dorsomedial part	2	16				
Retroambiguus nucleus	2	17				
Ventromedial preoptic nucleus	2	26				
