# Supplementary material for: Connectome-based prediction of functional impairment in experimental stroke models
Source: PLoS One. 2024 Dec 19;19(12):e0310743. doi: 10.1371/journal.pone.0310743 (PMC11658581; doi:10.1371/journal.pone.0310743)
Supplement: S8 Table — The marker column contains the code “3” for regions involved in learning and “2” for regions involved in motor behavior. The lesioned regions are shown in the columns PtA_R, AID_R, AIV_R, DI_R, GI_R, S1_R. Dout and Din are indicating the minimal graph theoretical distance to link a functional region and a lesioned regions (columns 3, 8, 13, 18…). Dspat is the spatial distance between a pair of regions. CMIAll are the pairwise values for the similarity of connections of a functional region and a lesioned region. FHN are pairwise coactivations of a functional region and a lesioned region. The regions were sorted by their functional groups (marker column). (PDF) [file pone.0310743.s014.pdf]

S7 Table. The similarity of functionally defined regions listed in the first column and the 6 lesioned regions of the dMCAO model. The marker column contains the code “3” for regions involved in learning and “2” for regions involved in motor behavior. The lesioned regions are shown in the columns PtA_R, AID_R, AIV_R, DI_R, GI_R, S1_R. Dout and Din are indicating the minimal graph theoretical distance to link a functional region and a lesioned regions (columns 3, 8, 13, 18...). Dspat is the spatial distance between a pair of regions. CMIAll are the pairwise values for the similarity of connections of a functional region and a lesioned region. FHN are pairwise coactivations of a functional region and a lesioned region. The regions were sorted by their functional groups (marker column).

	Marker	PtA_R D_{out}	D_{in}	D_{spat}	CMI All	FHN-Si...	AID_R D_{out}	D_{in}	D_{spat}	CMI All	FHN-Si...	AIV_R D_{out}	D_{in}	D_{spat}	CMI All	FHN-Si...	DI_R D_{out}	D_{in}	D_{spat}	CMI All	FHN-...	GI_R D_{out}	D_{in}	D_{spat}	CMI All	FHN-Si...	S1_R D_{out}	D_{in}	D_{spat}	CMI All	FHN-Simul...
Subparafascicular_thalamic_nucleus_rostral_part_R	3	2	2	-1	0,222	0,172	2	2	-1	0,295	0,151	2	2	-1	0,212	0,452	2	2	-1	0,265	0,699	2	2	-1	0,307	0,667	2	2	-1	0,207	0,559
Field_CA2_of_hippocampus_R	3	2	2	535,195	0,237	0,158	2	2	932,376	0,25	0,139	2	2	936,18	0,279	0,416	2	2	699,028	0,171	0,644	2	2	702,725	0,217	0,614	2	2	592,472	0,158	0,485
Posterior_thalamic_nuclear_group_R	3	1	1	701,083	0,365	0,182	2	2	1.029,078	0,305	0,159	2	2	1.022,349	0,267	0,477	2	1	812,314	0,383	0,739	1	1	822,204	0,403	0,705	1	1	796,239	0,357	0,591
Field_CA1_of_hippocampus_R	3	1	2	711,294	0,379	0,356	1	2	1.445,085	0,429	0,311	1	2	1.451,483	0,459	0,776	1	2	1.143,747	0,364	0,642	1	2	1.120,634	0,404	0,672	2	2	621,444	0,314	0,865
Dentate_gyrus_R	3	2	2	714,193	0,341	0,2	2	2	1.018,62	0,265	0,175	2	2	1.010,923	0,31	0,525	2	2	781,459	0,19	0,812	2	2	787,012	0,238	0,775	2	2	772,876	0,221	0,65
Field_CA3_of_hippocampus_R	3	2	1	726,253	0,299	0,225	2	2	964,002	0,308	0,197	2	2	955,658	0,284	0,592	2	2	724,846	0,174	0,915	2	2	730,617	0,242	0,873	2	2	767,164	0,2	0,732
Presubiculum_R	3	1	1	791,948	0,362	0,235	2	2	1.452,494	0,328	0,206	2	1	1.449,575	0,319	0,618	2	2	1.166,649	0,27	0,956	2	1	1.154,372	0,293	0,912	2	2	809,401	0,292	0,714
Postrhinal_cortex_R	3	1	1	827,944	0,425	0,203	1	1	1.767,884	0,376	0,23	1	1	1.777,661	0,377	0,689	1	1	1.477,709	0,314	0,881	2	1	1.456,632	0,353	0,922	1	1	814,13	0,328	0,712
Parasubiculum_R	3	1	1	863,457	0,346	0,281	2	2	1.612,44	0,42	0,246	2	1	1.611,779	0,396	0,737	2	2	1.319,983	0,281	0,877	2	1	1.304,087	0,274	0,919	2	2	870,038	0,262	0,847
Perirhinal_cortex_R	3	1	1	870,003	0,588	0,062	1	1	1.255,207	0,571	0,071	1	1	1.255,758	0,541	0,024	1	1	936,004	0,5	0,015	1	1	904,847	0,512	0,016	1	1	681,628	0,509	0,019
Lateral_entorhinal_cortex_R	3	1	1	962,678	0,462	0,062	1	1	1.352,401	0,531	0,071	1	1	1.348,996	0,558	0,024	1	1	1.032,902	0,43	0,015	1	1	1.003,239	0,444	0,016	1	1	797,416	0,393	0,019
Subiculum_R	3	1	2	1.019,032	0,338	0,533	1	2	1.453,533	0,446	0,467	2	1	1.439,099	0,462	0,6	2	2	1.264,655	0,413	0,462	2	2	1.278,486	0,381	0,484	2	2	1.189,659	0,283	0,577
Cingulate_cortex_area_2_R	3	1	2	1.037,961	0,417	0,232	1	1	798,07	0,414	0,203	2	2	812,31	0,398	0,609	2	2	896,551	0,369	0,942	2	2	938,445	0,398	0,899	1	1	1.183,578	0,384	0,729
Cingulate_cortex_area_1_R	3	1	1	1.070,919	0,482	0,32	1	2	879,819	0,527	0,28	2	2	903,8	0,456	0,84	2	2	1.000,448	0,427	0,769	2	2	1.039,474	0,427	0,806	1	1	1.221,465	0,467	0,962
Reuniens_thalamic_nucleus_R	3	1	1	1.100,121	0,352	0,062	1	1	975,581	0,412	0,071	1	1	951,441	0,365	0,024	1	2	888,827	0,358	0,015	1	2	921,385	0,375	0,016	1	1	1.218,618	0,332	0,019
Rhomboid_thalamic_nucleus_R	3	1	1	1.113,809	0,347	0,308	1	2	1.001,801	0,391	0,269	1	1	975,963	0,379	0,774	1	2	899,542	0,354	0,696	1	2	929,886	0,408	0,727	1	1	1.224,517	0,354	0,926
Interoanteromedial_thalamic_nucleus_R	3	2	2	1.122,136	0,32	0,147	2	2	962,356	0,304	0,128	2	2	939,04	0,338	0,385	1	2	901,471	0,286	0,596	2	2	937,057	0,341	0,569	2	2	1.249,212	0,269	0,45
Lateral_globus_pallidus_R	2	2	2	-1	0,286	0,144	2	2	-1	0,174	0,126	2	2	-1	0,187	0,378	2	2	-1	0,162	0,586	2	2	-1	0,213	0,559	2	2	-1	0,225	0,468
Lateral_agranular_prefrontal_cortex_R	2	1	1	787,125	0,625	0,438	1	1	682,91	0,45	0,312	1	1	722,792	0,421	0,114	1	1	723,666	0,414	0,108	1	1	749,69	0,476	0,113	1	1	856,534	0,71	0,135
Medial_agranular_prefrontal_cortex_R	2	1	1	842,936	0,595	0,125	1	1	737,511	0,534	0,067	1	2	768,423	0,503	0,023	1	1	796,303	0,465	0,031	1	1	829,045	0,49	0,032	1	1	960,639	0,628	0,038
Ventrolateral_thalamic_nucleus_R	2	1	1	874,354	0,377	0,235	2	2	820,59	0,327	0,206	2	1	806,173	0,236	0,618	2	2	682,051	0,271	0,9	2	2	709,259	0,319	0,912	1	1	961,443	0,391	0,765
Caudate_putamen_R	2	1	1	937,977	0,523	0,062	2	1	441,872	0,48	0,071	2	1	429,547	0,374	0,024	2	1	291,753	0,408	0,015	2	1	327,868	0,455	0,016	1	1	904,708	0,491	0,019
Cerebellar_nuclei_R	2	2	2	992,999	0,315	0,14	2	2	1.667,505	0,12	0,123	2	2	1.660,004	0,117	0,368	2	2	1.392,824	0,192	0,57	2	2	1.384,652	0,232	0,544	2	1	1.058,147	0,299	0,456
Medial_globus_pallidus_R	2	1	1	1.076,331	0,303	0,17	2	2	812,71	0,23	0,149	2	2	782,21	0,265	0,447	2	2	614,886	0,241	0,691	2	2	629,925	0,291	0,66	1	2	1.061,719	0,288	0,553
Subthalamic_nucleus_R	2	1	1	1.077,066	0,358	0,444	2	1	1.030,705	0,393	0,389	1	1	1.003,352	0,322	0,625	2	1	805,656	0,38	0,485	2	1	813,016	0,387	0,508	1	1	1.079,598	0,394	0,66
Substantia_nigra_compact_part_R	2	1	1	1.120,37	0,377	0,281	2	2	1.361,203	0,321	0,246	2	2	1.338,339	0,326	0,737	2	1	1.129,666	0,331	0,794	1	1	1.134,593	0,348	0,831	1	1	1.180,405	0,4	0,912
Substantia_nigra_reticular_part_R	2	2	1	1.120,901	0,376	0,2	2	1	1.346,247	0,263	0,175	2	2	1.323,076	0,317	0,525	2	2	1.110,221	0,321	0,812	2	1	1.114,001	0,374	0,775	2	1	1.169,922	0,438	0,65
Cerebellar_cortex_R	2	2	1	1.269,608	0,159	0,216	2	2	2.278,677	0,121	0,189	2	2	2.284,045	0,1	0,547	3	2	2.001,848	0,122	0,738	2	2	1.987,616	0,13	0,744	2	1	1.359,344	0,164	0,68
Pontine_nuclei_R	2	2	1	1.574,822	0,223	0,152	2	2	1.843,015	0,199	0,133	2	1	1.814,201	0,146	0,4	2	2	1.607,392	0,211	0,619	2	1	1.608,268	0,266	0,59	1	1	1.626,726	0,259	0,495
