# Supplementary material for: Connectome-based prediction of functional impairment in experimental stroke models
Source: PLoS One. 2024 Dec 19;19(12):e0310743. doi: 10.1371/journal.pone.0310743 (PMC11658581; doi:10.1371/journal.pone.0310743)
Supplement: S10 Table — The articles which provides evidence for at least one of these functions are quoted in the column References. Sorting was performed for CMIAll ranks. Marker 0 indicates regions of the control connectome. Those with large ranks were shown, only. The average rank column displays the average rank values of the local parameter computation. (PDF) [file pone.0310743.s016.pdf]

S9 Table. Ranks of lesioned regions of the dMCAO experiment and the motor (Marker: 2) as well learning behavior (Marker: 3) groups. The articles which provides evidence for at least one of these functions are quoted in the column References. Sorting was performed for CMI_{All} ranks. Marker 0 indicates regions of the control connectome. Those with large ranks were shown, only. The average rank column displays the average rank values of the local parameter computation.

Control connectome region	Marker	References	CMI_{All} rank	Lesioned region	FHN rank	Lesioned region	Average rank
Perirhinal cortex	3	[1, 2, 3, 4]	5	Parietal association cortex	13	Agranular insular cortex dorsal part	30
Cingulate cortex area 1	3	[5, 6, 7]	6	Agranular insular cortex dorsal part	2	Primary somatosensory cortex	29
Lateral entorhinal cortex	3	[8]	11	Agranular insular cortex ventral part	13	Agranular insular cortex dorsal part	13
Cingulate cortex area 2	3	[5, 6, 7]	11	Parietal association cortex	9	Dysgranular insular cortex	55
Posterior thalamic nuclear group	3	[9]	12	Granular insular cortex	90	Dysgranular insular cortex	97
Parasubiculum	3	[10]	15	Agranular insular cortex dorsal part	9	Granular insular cortex	88
Anteroventral thalamic nucleus	3	[11, 12, 13]	17	Parietal association cortex	213	Dysgranular insular cortex	119
Interoanteromedial thalamic nucleus	3	[14, 15, 16, 17]	17	Granular insular cortex	228	Dysgranular insular cortex	178
Subiculum	3	[18, 19]	18	Agranular insular cortex ventral part	23	Agranular insular cortex ventral part	46
Dentate gyrus	3	[20, 21, 22]	19	Parietal association cortex	47	Dysgranular insular cortex	89
Subparafascicular thalamic nucleus rostral part	3	[23, 24]	20	Granular insular cortex	207	Dysgranular insular cortex	132
Postrhinal cortex	3	[25, 26, 27]	20	Parietal association cortex	5	Granular insular cortex	100
Field CA1 of hippocampus	3	[28, 29, 30]	23	Agranular insular cortex ventral part	6	Primary somatosensory cortex	63
Presubiculum	3	[31, 32]	25	Parietal association cortex	7	Dysgranular insular cortex	81
Anterodorsal thalamic nucleus	3	[33, 34]	30	Parietal association cortex	265	Dysgranular insular cortex	153
Field CA2 of hippocampus	3	[35, 36]	33	Agranular insular cortex ventral part	215	Dysgranular insular cortex	161
Rhomboid thalamic nucleus	3	[37, 38, 39]	36	Granular insular cortex	4	Primary somatosensory cortex	40
Reuniens thalamic nucleus	3	[37, 38, 39]	40	Agranular insular cortex dorsal part	13	Agranular insular cortex dorsal part	10
Field CA3 of hippocampus	3	[8, 40, 41]	41	Agranular insular cortex dorsal part	14	Dysgranular insular cortex	109
Mammillary body	3	[42, 43, 44, 45]	63	Parietal association cortex	13	Agranular insular cortex dorsal part	17
Lateral agranular prefrontal cortex	2	[46, 47]	2	Primary somatosensory cortex	4	Parietal association cortex	6
Medial agranular prefrontal cortex	2	[48, 49, 50]	2	Primary somatosensory cortex	13	Parietal association cortex	5
Caudate putamen	2	[51, 52]	5	Parietal association cortex	13	Agranular insular cortex dorsal part	44
Substantia nigra reticular part	2	[53, 54]	9	Primary somatosensory cortex	49	Dysgranular insular cortex	53
Lateral globus pallidus	2	[55, 56]	11	Parietal association cortex	248	Dysgranular insular cortex	184
Ventrolateral thalamic nucleus	2	[57]	15	Primary somatosensory cortex	14	Granular insular cortex	80
Cerebellar nuclei	2	[58, 59, 60]	25	Parietal association cortex	280	Dysgranular insular cortex	118
Substantia nigra compact part	2	[61, 62]	27	Primary somatosensory cortex	5	Primary somatosensory cortex	31
Medial globus pallidus	2	[61, 63, 64]	27	Parietal association cortex	207	Dysgranular insular cortex	85
Pontine nuclei	2	[65, 66, 67, 68, 69]	29	Granular insular cortex	256	Dysgranular insular cortex	108
Subthalamic nucleus	2	[56, 70, 71]	33	Primary somatosensory cortex	24	Primary somatosensory cortex	54
Cerebellar cortex	2	[58, 72, 73, 74]	91	Primary somatosensory cortex	37	Granular insular cortex	182
Agranular insular cortex posterior part	0		1	Agranular insular cortex ventral part	7	Agranular insular cortex ventral part	47
Secondary somatosensory cortex	0		1	Primary somatosensory cortex	2	Granular insular cortex	58
Primary visual cortex	0		1	Parietal association cortex	7	Granular insular cortex	67
Anterior basolateral nucleus	0		2	Agranular insular cortex dorsal part	8	Primary somatosensory cortex	57
Frontal cortex area 3	0		3	Agranular insular cortex ventral part	225	Dysgranular insular cortex	197
Secondary visual cortex lateral area	0		3	Parietal association cortex	244	Dysgranular insular cortex	133
Lateral orbital cortex	0		3	Agranular insular cortex dorsal part	14	Parietal association cortex	34
Ventral orbital cortex	0		3	Agranular insular cortex dorsal part	7	Parietal association cortex	21
Dorsal striatum	0		4	Parietal association cortex	9	Granular insular cortex	143
Temporal association cortex 1	0		4	Parietal association cortex	233	Dysgranular insular cortex	154
Dorsal peduncular cortex	0		4	Agranular insular cortex ventral part	254	Dysgranular insular cortex	159
Dorsolateral orbital cortex	0		4	Agranular insular cortex dorsal part	234	Dysgranular insular cortex	172
Medial orbital cortex	0		4	Agranular insular cortex dorsal part	16	Agranular insular cortex ventral part	22
