# Supplementary material for: Connectome-based prediction of functional impairment in experimental stroke models
Source: PLoS One. 2024 Dec 19;19(12):e0310743. doi: 10.1371/journal.pone.0310743 (PMC11658581; doi:10.1371/journal.pone.0310743)
Supplement: S11 Table — Sorting was performed for the FHN rank computed with the coactivation matrix of a FHN simulation. The average rank column displays the average rank values of the local parameter computation. (PDF) [file pone.0310743.s017.pdf]

**S10 Table. Ranks of lesioned regions of the dMCAO experiment and the motor (Marker: 2) as well learning behavior (Marker: 3) groups.** Sorting was performed for the FHN rank computed with the coactivation matrix of a FHN simulation. The average rank column displays the average rank values of the local parameter computation.

| Control connectome region | Marker | $CMI_{All}$ rank | Lesioned region | FHN rank | Lesioned region | Average rank |
|---|---|---|---|---|---|---|
| Cingulate cortex area 1 | 3 | 6 | Agranular insular cortex dorsal part | 2 | Primary somatosensory cortex | 29 |
| Rhomboid thalamic nucleus | 3 | 36 | Granular insular cortex | 4 | Primary somatosensory cortex | 40 |
| Postrhinal cortex | 3 | 20 | Parietal association cortex | 5 | Granular insular cortex | 100 |
| Field CA1 of hippocampus | 3 | 23 | Agranular insular cortex ventral part | 6 | Primary somatosensory cortex | 63 |
| Presubiculum | 3 | 25 | Parietal association cortex | 7 | Dysgranular insular cortex | 81 |
| Cingulate cortex area 2 | 3 | 11 | Parietal association cortex | 9 | Dysgranular insular cortex | 55 |
| Parasubiculum | 3 | 15 | Agranular insular cortex dorsal part | 9 | Granular insular cortex | 88 |
| Perirhinal cortex | 3 | 5 | Parietal association cortex | 13 | Agranular insular cortex dorsal part | 30 |
| Lateral entorhinal cortex | 3 | 11 | Agranular insular cortex ventral part | 13 | Agranular insular cortex dorsal part | 13 |
| Reuniens thalamic nucleus | 3 | 40 | Agranular insular cortex dorsal part | 13 | Agranular insular cortex dorsal part | 10 |
| Mammillary body | 3 | 63 | Parietal association cortex | 13 | Agranular insular cortex dorsal part | 17 |
| Field CA3 of hippocampus | 3 | 41 | Agranular insular cortex dorsal part | 14 | Dysgranular insular cortex | 109 |
| Subiculum | 3 | 18 | Agranular insular cortex ventral part | 23 | Agranular insular cortex ventral part | 46 |
| Dentate gyrus | 3 | 19 | Parietal association cortex | 47 | Dysgranular insular cortex | 89 |
| Posterior thalamic nuclear group | 3 | 12 | Granular insular cortex | 90 | Dysgranular insular cortex | 97 |
| Subparafascicular thalamic nucleus rostral part | 3 | 20 | Granular insular cortex | 207 | Dysgranular insular cortex | 132 |
| Anteroventral thalamic nucleus | 3 | 17 | Parietal association cortex | 213 | Dysgranular insular cortex | 119 |
| Field CA2 of hippocampus | 3 | 33 | Agranular insular cortex ventral part | 215 | Dysgranular insular cortex | 161 |
| Interoanteromedial thalamic nucleus | 3 | 17 | Granular insular cortex | 228 | Dysgranular insular cortex | 178 |
| Anterodorsal thalamic nucleus | 3 | 30 | Parietal association cortex | 265 | Dysgranular insular cortex | 153 |
| Lateral agranular prefrontal cortex | 2 | 2 | Primary somatosensory cortex | 4 | Parietal association cortex | 6 |
| Substantia nigra compact part | 2 | 27 | Primary somatosensory cortex | 5 | Primary somatosensory cortex | 31 |
| Medial agranular prefrontal cortex | 2 | 2 | Primary somatosensory cortex | 13 | Parietal association cortex | 5 |
| Caudate putamen | 2 | 5 | Parietal association cortex | 13 | Agranular insular cortex dorsal part | 44 |
| Ventrolateral thalamic nucleus | 2 | 15 | Primary somatosensory cortex | 14 | Granular insular cortex | 80 |
| Subthalamic nucleus | 2 | 33 | Primary somatosensory cortex | 24 | Primary somatosensory cortex | 54 |
| Cerebellar cortex | 2 | 91 | Primary somatosensory cortex | 37 | Granular insular cortex | 182 |
| Substantia nigra reticular part | 2 | 9 | Primary somatosensory cortex | 49 | Dysgranular insular cortex | 53 |
| Medial globus pallidus | 2 | 27 | Parietal association cortex | 207 | Dysgranular insular cortex | 85 |
| Lateral globus pallidus | 2 | 11 | Parietal association cortex | 248 | Dysgranular insular cortex | 184 |
| Pontine nuclei | 2 | 29 | Granular insular cortex | 256 | Dysgranular insular cortex | 108 |
| Cerebellar nuclei | 2 | 25 | Parietal association cortex | 280 | Dysgranular insular cortex | 118 |
| Secondary somatosensory cortex | 0 | 1 | Primary somatosensory cortex | 2 | Granular insular cortex | 58 |
| Agranular insular cortex posterior part | 0 | 1 | Agranular insular cortex ventral part | 7 | Agranular insular cortex ventral part | 47 |
| Primary visual cortex | 0 | 1 | Parietal association cortex | 7 | Granular insular cortex | 67 |
| Ventral orbital cortex | 0 | 3 | Agranular insular cortex dorsal part | 7 | Parietal association cortex | 21 |
| Anterior basolateral nucleus | 0 | 2 | Agranular insular cortex dorsal part | 8 | Primary somatosensory cortex | 57 |
| Dorsal striatum | 0 | 4 | Parietal association cortex | 9 | Granular insular cortex | 143 |
| Lateral orbital cortex | 0 | 3 | Agranular insular cortex dorsal part | 14 | Parietal association cortex | 34 |
| Medial orbital cortex | 0 | 4 | Agranular insular cortex dorsal part | 16 | Agranular insular cortex ventral part | 22 |
| Frontal cortex area 3 | 0 | 3 | Agranular insular cortex ventral part | 225 | Dysgranular insular cortex | 197 |
| Temporal association cortex 1 | 0 | 4 | Parietal association cortex | 233 | Dysgranular insular cortex | 154 |
| Dorsolateral orbital cortex | 0 | 4 | Agranular insular cortex dorsal part | 234 | Dysgranular insular cortex | 172 |
| Secondary visual cortex lateral area | 0 | 3 | Parietal association cortex | 244 | Dysgranular insular cortex | 133 |
| Dorsal peduncular cortex | 0 | 4 | Agranular insular cortex ventral part | 254 | Dysgranular insular cortex | 159 |
