# Supplementary material for: Connectome-based prediction of functional impairment in experimental stroke models
Source: PLoS One. 2024 Dec 19;19(12):e0310743. doi: 10.1371/journal.pone.0310743 (PMC11658581; doi:10.1371/journal.pone.0310743)
Supplement: S12 Table — Sorting was performed for the CMIAll rank. (PDF) [file pone.0310743.s018.pdf]

S11 Table. Ranks of lesioned regions of the ICH experiment and the motor (Marker: 2) as well learning behavior (Marker: 3) groups. Sorting was performed for the CMI_{All} rank.

Control connectome region	Marker	CMI_{All} rank	Lesioned region	FHN rank	Lesioned region	Average rank
Perirhinal cortex	3	3	Basolateral amygdaloid nucleus	1	Reticular thalamic nucleus	27
Lateral entorhinal cortex	3	4	Basolateral amygdaloid nucleus	1	Reticular thalamic nucleus	9
Rhomboid thalamic nucleus	3	5	Paracentral thalamic nucleus	2	Ventromedial thalamic nucleus	30
Subiculum	3	5	Basolateral amygdaloid nucleus	2	Endopiriform system	41
Field CA1 of hippocampus	3	7	Basolateral amygdaloid nucleus	2	Central amygdaloid nucleus medial division	61
Field CA2 of hippocampus	3	7	Endopiriform system	13	Anteroventral thalamic nucleus	147
Presubiculum	3	7	Anteroventral thalamic nucleus	5	Basal nucleus Meynert	84
Mammillary body	3	10	Central amygdaloid nucleus	1	Reticular thalamic nucleus	28
Dentate gyrus	3	11	Anteroventral thalamic nucleus	2	Medial globus pallidus	102
Parasubiculum	3	11	Anteroventral thalamic nucleus	11	Interstitial nucleus of the posterior limb of the anterior commissure	87
Cingulate cortex area 1	3	11	Caudate putamen	11	Interstitial nucleus of the posterior limb of the anterior commissure	24
Cingulate cortex area 2	3	12	Basal nucleus Meynert	3	Basal nucleus Meynert	63
Field CA3 of hippocampus	3	15	Endopiriform system	11	Paracentral thalamic nucleus	104
Postrhinal cortex	3	16	Anteroventral thalamic nucleus	13	Basal nucleus Meynert	98
Subparafascicular thalamic nucleus rostral part	3	46	Basolateral amygdaloid nucleus	9	Mediodorsal thalamic nucleus lateral part	134
Ventrolateral thalamic nucleus	2	1	Ventromedial thalamic nucleus	4	Basal nucleus Meynert	89
Pontine nuclei	2	2	Centrolateral thalamic nucleus	32	Anteroventral thalamic nucleus	108
Subthalamic nucleus	2	2	Caudate putamen	3	Endopiriform system	55
Cerebellar cortex	2	3	Ventrobasal complex	2	Paracentral thalamic nucleus	180
Lateral agranular prefrontal cortex	2	4	Caudate putamen	3	Caudate putamen	4
Substantia nigra reticular part	2	5	Medial globus pallidus	10	Mediodorsal thalamic nucleus lateral part	51
Medial agranular prefrontal cortex	2	10	Caudate putamen	1	Reticular thalamic nucleus	1
Substantia nigra compact part	2	17	Basolateral amygdaloid nucleus	7	Interstitial nucleus of the posterior limb of the anterior commissure	35
Cerebellar nuclei	2	21	Ventrobasal complex	31	Ventro anterior thalamic nucleus	120
Lateral hypothalamic area	0	1	Central amygdaloid nucleus	1	Reticular thalamic nucleus	45
Bed nucleus of the stria terminalis intraamygdaloid division	0	1	Bed nucleus of the stria terminalis medial division	5	Mediodorsal thalamic nucleus lateral part	143
Bed nucleus of the stria terminalis lateral division ventral part	0	1	Bed nucleus of the stria terminalis medial division	3	Mediodorsal thalamic nucleus lateral part	100
Amygdaloid intramedullary gray	0	1	Amygdalostriatal transition area	89	Ventro anterior thalamic nucleus	393
Dorsal striatum	0	1	Caudate putamen	7	Laterodorsal thalamic nucleus	141
