# Supplementary material for: Connectome-based prediction of functional impairment in experimental stroke models
Source: PLoS One. 2024 Dec 19;19(12):e0310743. doi: 10.1371/journal.pone.0310743 (PMC11658581; doi:10.1371/journal.pone.0310743)
Supplement: S13 Table — Larger similarity values indicate a stronger similarity. The maximum values for each experimental group and method were highlighted. (PDF) [file pone.0310743.s019.pdf]

S12 Table. Similarities of lesioned regions with regard to motor regions or learning regions or non-lesioned regions. Larger similarity values indicate a stronger similarity. The maximum values for each experimental group and method were highlighted.

Method	Comparison	dMCAO	ICH	sMCAO
CMI_{All}	Lesioned vs motor	0.328	**0.271**	0.251
	Lesioned vs learning	**0.352**	0.256	**0.302**
	Lesioned vs non-lesioned	0.119	0.103	0.104
FHN-model	Lesioned vs motor	0.359	**0.399**	0.425
	Lesioned vs learning	**0.432**	0.384	**0.471**
	Lesioned vs non-lesioned	0.278	0.343	0.355
